# Supplementary figures and images for: Neural responses in macaque prefrontal cortex are linked to strategic exploration
Source: PLoS Biol. 2023 Jan 30;21(1):e3001985. doi: 10.1371/journal.pbio.3001985 (PMC9910800; doi:10.1371/journal.pbio.3001985)

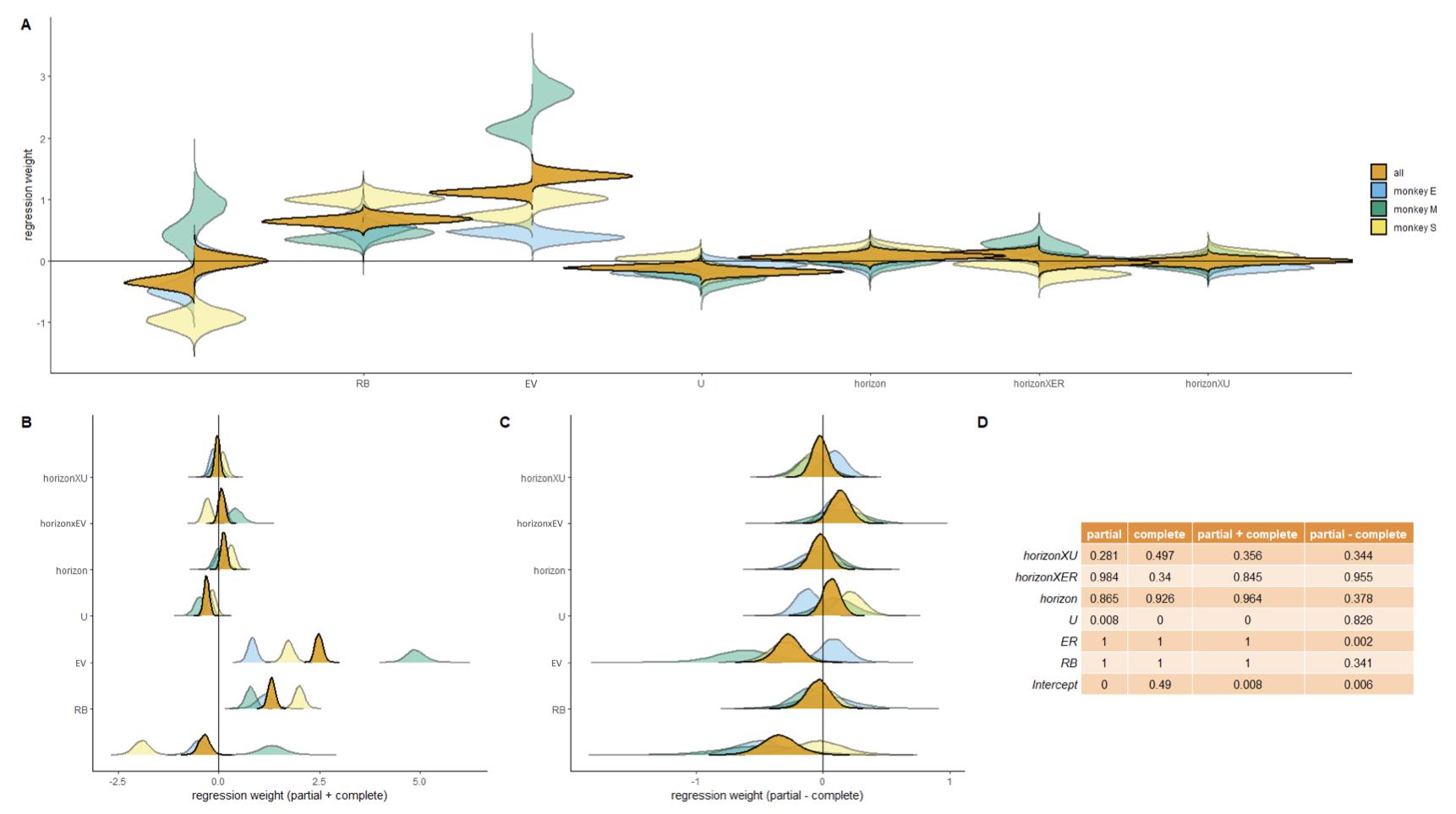

Supplement: S1 Fig — (A) Predictors are from left to right: Intercept (i.e., a side bias), repetition bias (RB), expected value of difference between right and left according to our Bayesian model (EV), uncertainty difference between right and left according to our Bayesian model (U), horizon length (short horizon is positive, long horizon is negative), the interaction between horizon and expected value (horizonXER), and the interaction between horizon and uncertainty (horizonXU). The distributions are the posteriors of the parameter estimates, shown both for each monkey individually and averaged over animals. Fits from the partial feedback sessions are shown on the left, and from the complete feedback sessions on the right. (B) Data from the same fit as in (A) but now summed up over both partial and complete feedback sessions. (C) Data from the same fit as in (A) but now we computed the difference between partial and complete feedback sessions. (D) One-sided p-values for all parameters are computed as the number of samples of the posterior greater than 0. To compute the p-value for effects smaller than 0, the p-values in the table can be subtracted from 1. Data and code to reproduce the figure can be found at https://doi.org/10.5281/zenodo.7464572. (TIFF) [file pbio.3001985.s001.tiff]

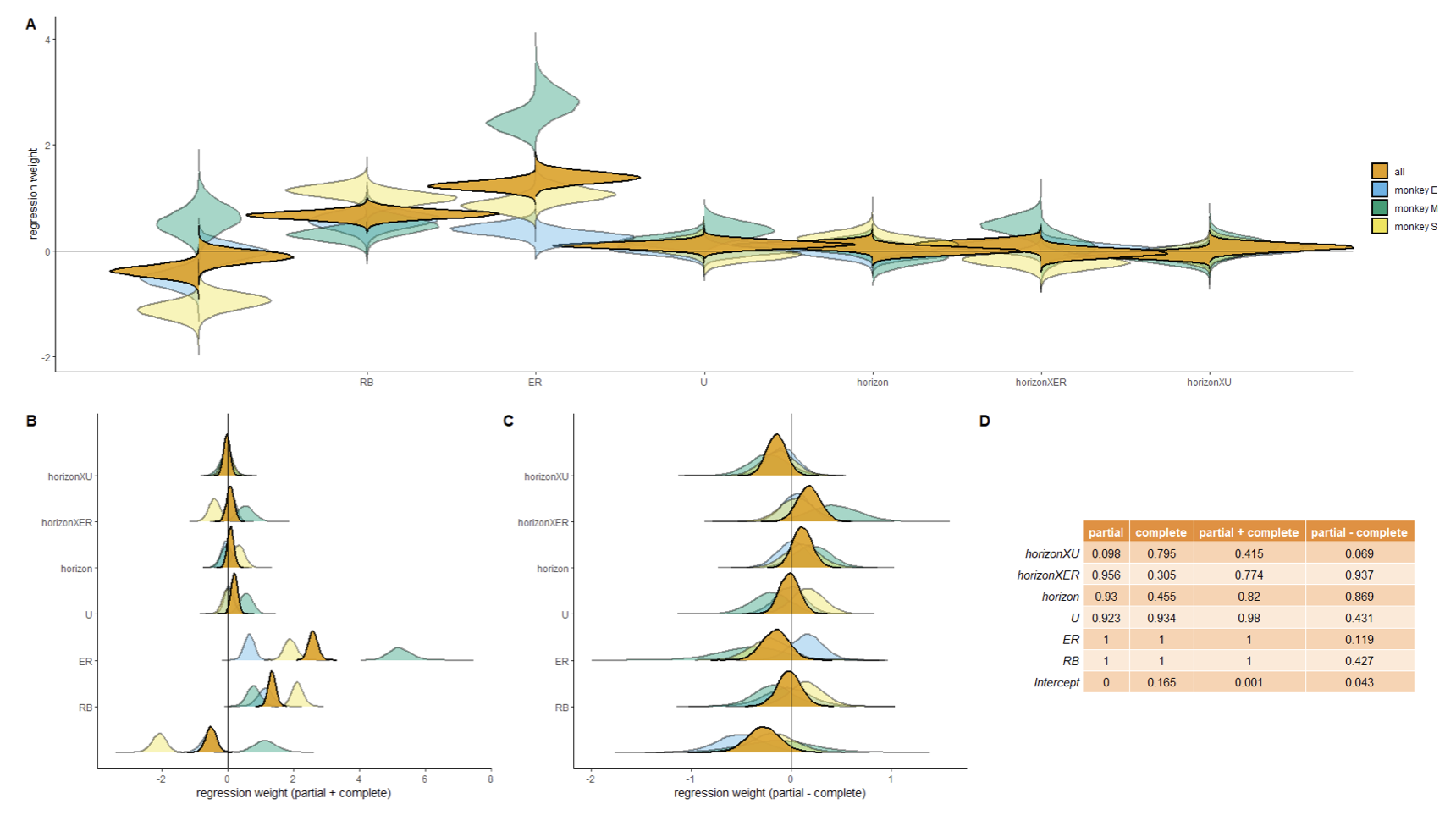

Supplement: S2 Fig — All conventions are the same as in S1 Fig. Data and code to reproduce the figure can be found at https://doi.org/10.5281/zenodo.7464572. (TIFF) [file pbio.3001985.s002.tiff]

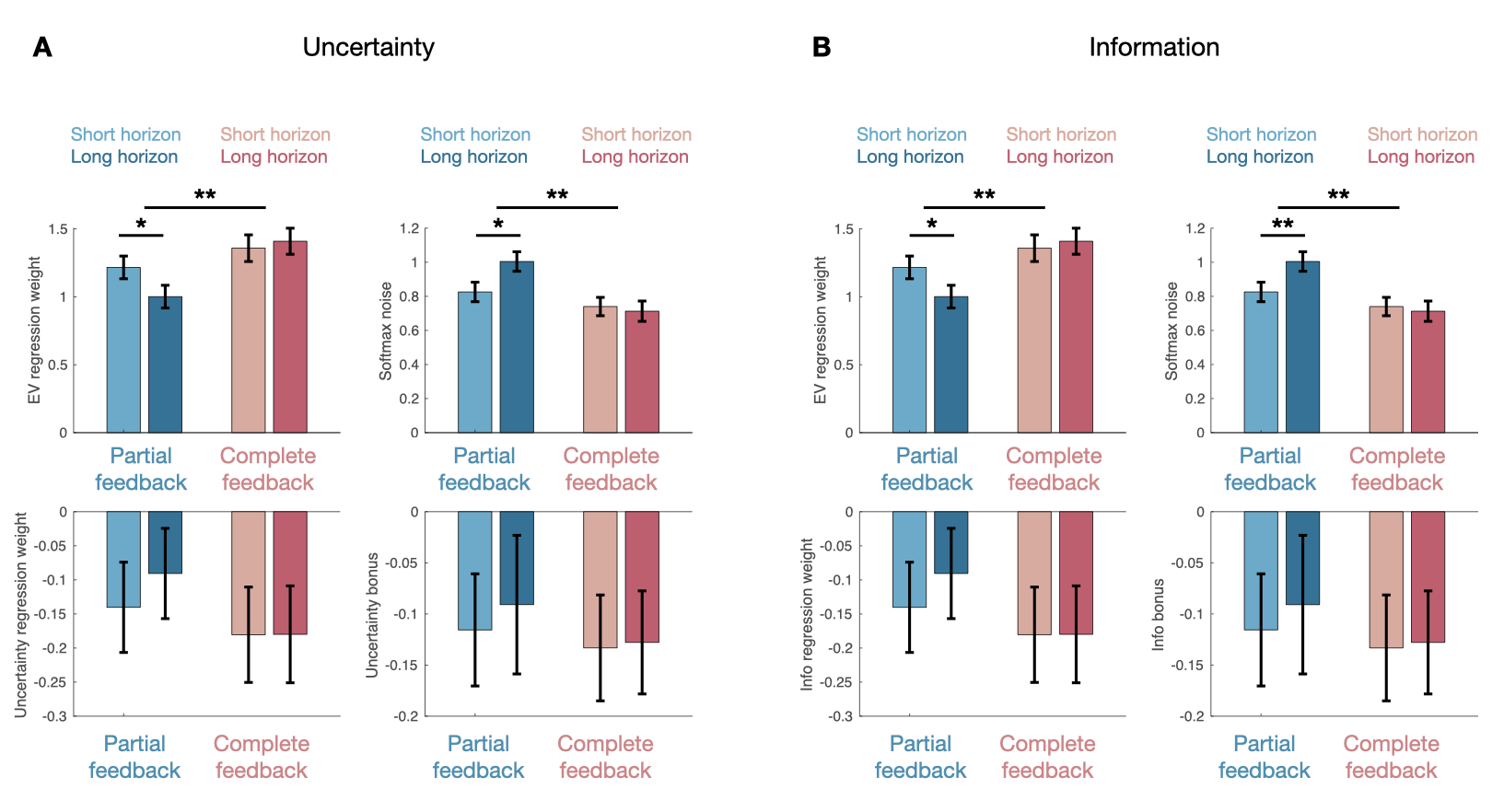

Supplement: S3 Fig — (A) With the uncertainty regressor. We find that monkeys modulate their sensitivity to the expected value depending on the horizon and the feedback type, which is equivalent to the random exploration parameter, the softmax noise, which is the inverse of the expected value regressor. However, we find no modulation of the uncertainty by the horizon nor the feedback type, which is equivalent to the directed exploration parameter, the uncertainty bonus, which is the uncertainty regressor divided by the expected value regressor. (B) Same as A but with the number of available information rather than the uncertainty. Error bars indicate standard deviation. Data and code to reproduce the figure can be found at https://doi.org/10.5281/zenodo.7464572. (TIFF) [file pbio.3001985.s003.tiff]

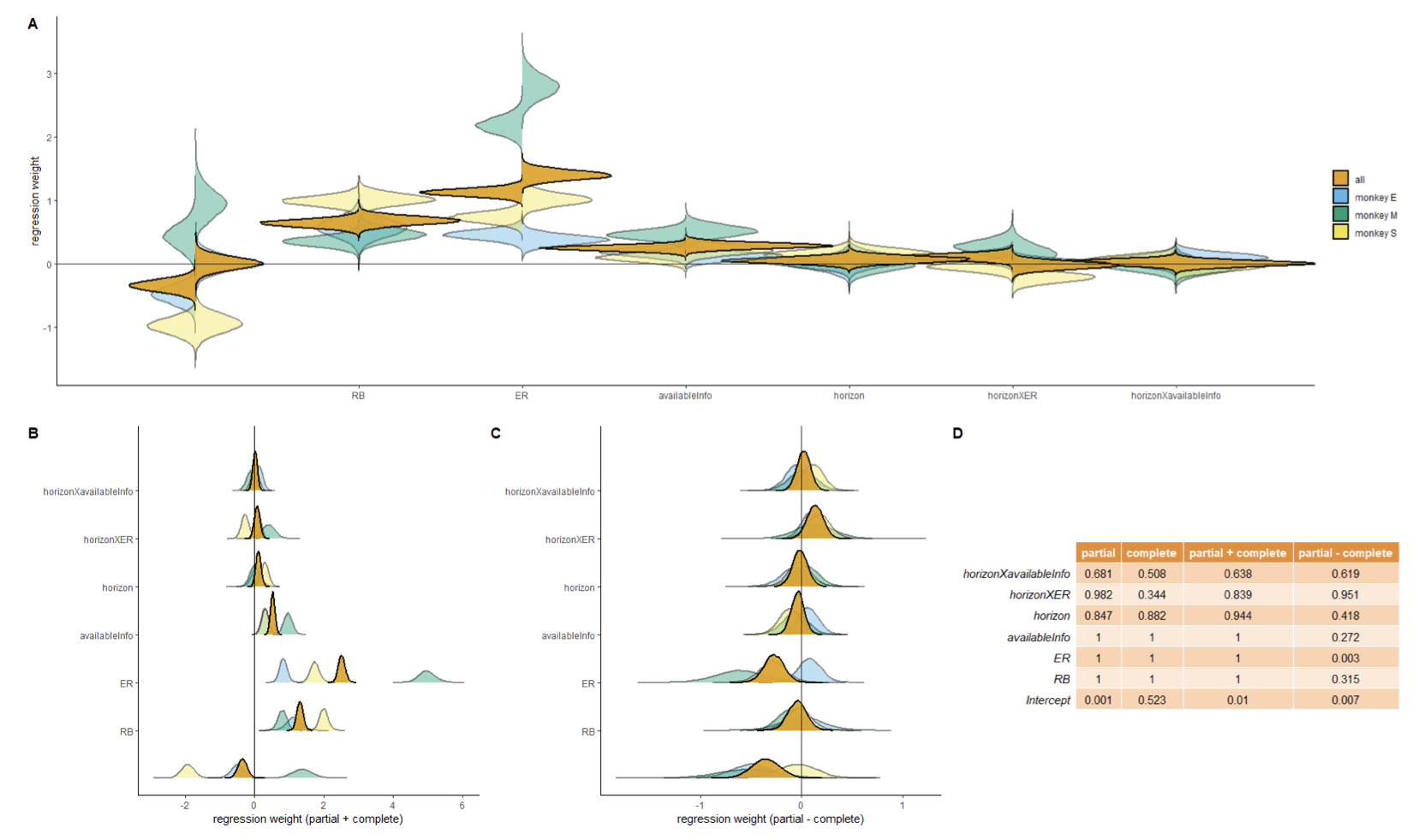

Supplement: S4 Fig — All conventions are the same as in S1 Fig. Data and code to reproduce the figure can be found at https://doi.org/10.5281/zenodo.7464572. (TIFF) [file pbio.3001985.s004.tiff]

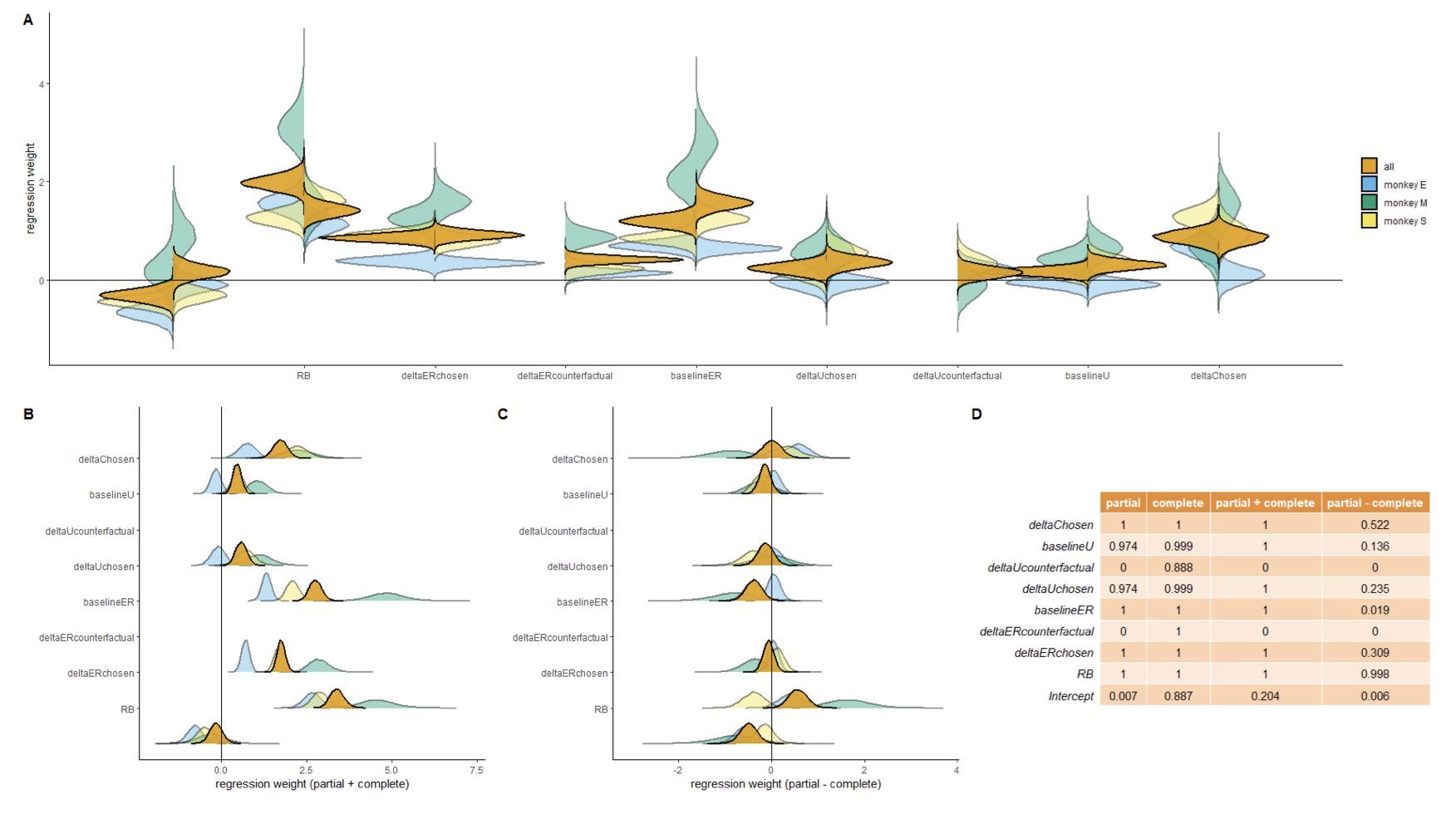

Supplement: S5 Fig — (A) Predictors are from left to right: Intercept (i.e., a side bias), repetition bias (RB), the change in expected value between the right and left option revealed by choices made during this horizon, compared to the initial expected value for this horizon, i.e., the baseline (deltaERchosen), the change in expected value between the right and left option revealed by feedback about the unchosen option, compared to the initial expected value for this horizon (deltaERcounterfactual), the difference in initial expected value between the right and left option available, i.e., the expected value difference at first choice (baselineU), the change in uncertainty between the right and left option revealed by choices made during this horizon, compared to the initial uncertainty for this horizon (deltaUchosen), the change in uncertainty between the right and left option revealed by feedback about the unchosen option, compared to the initial uncertainty for this horizon (deltaUcounterfactual), the difference in initial uncertainty between the right and left option available, i.e., the uncertainty difference at first choice (baselineU), the difference between how often the right option has been chosen over the left option during this horizon (deltaChosen). All other conventions are the same as in S1 Fig, also for panels B-D. Data and code to reproduce the figure can be found at https://doi.org/10.5281/zenodo.7464572. (TIFF) [file pbio.3001985.s005.tiff]

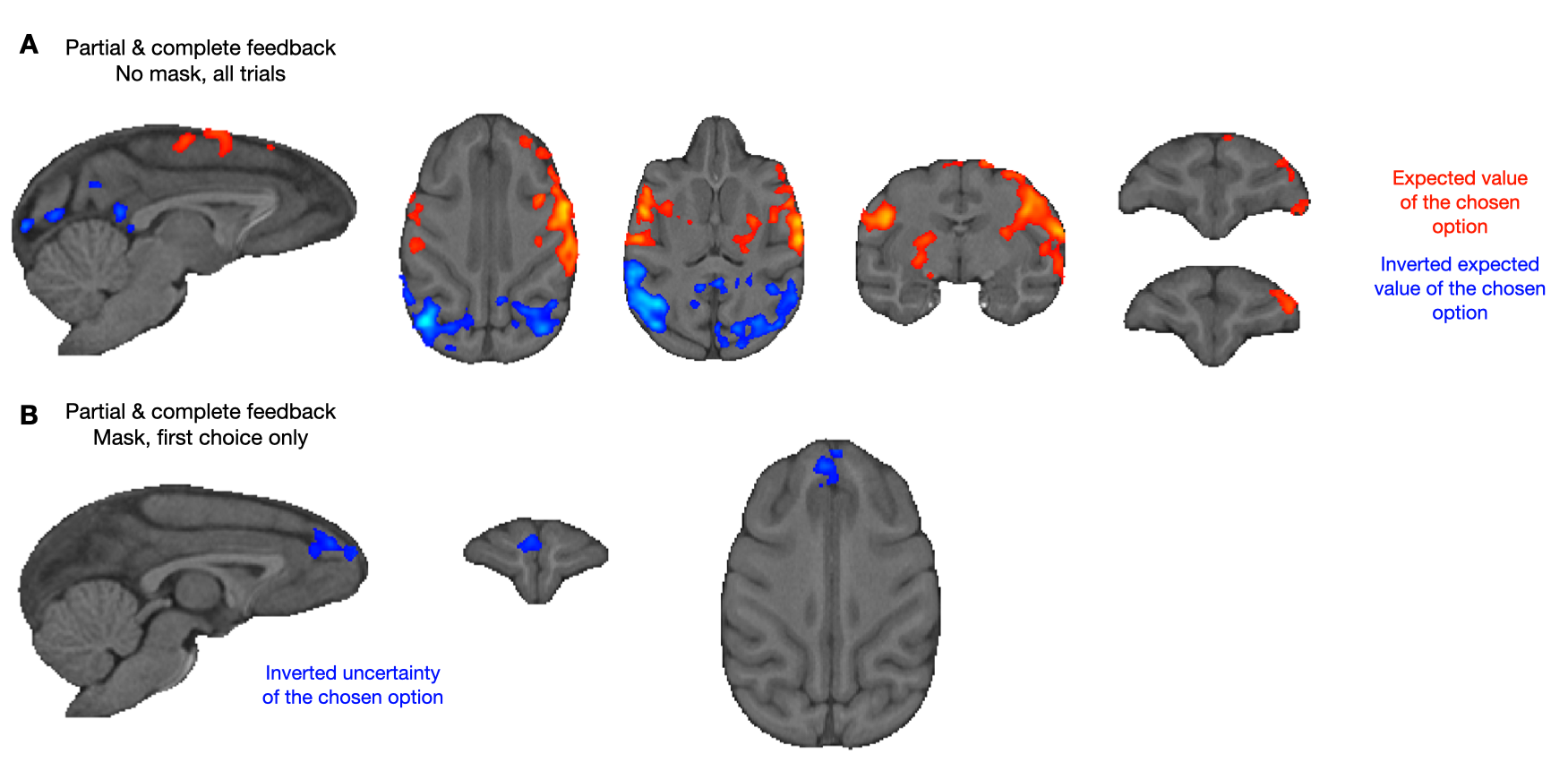

Supplement: S6 Fig — (A) Expected value of the chosen option without mask and when taking the activity before the choice in all trials (not just first choice trials), we observed large activations related to the expected value of the chosen option (which is the same as the chosen action in our task) spanning from the motor cortex/somatosensory cortex, the dlPFC, the OFC, and striatum, as well as an inverted signal in the visual areas (Cluster p < 0.05, cluster forming threshold of z > 2.3). (B) In the partial and complete feedback conditions in our VOI and when taking the activity before the first choice only, we found 1 cluster of activity related to the inverse of the magnitude of the uncertainty about the chosen option in the right medial prefrontal cortex (24c and 9m) that extended bilaterally in the frontal pole (10mr) (Cluster P < 0.05, cluster forming threshold of z > 2.3). Data to reproduce the figure can be found at https://doi.org/10.5281/zenodo.7464572. dlPFC, dorsolateral prefrontal cortex; OFC, orbitofrontal cortex; VOI, volume of interest. (TIFF) [file pbio.3001985.s006.tiff]

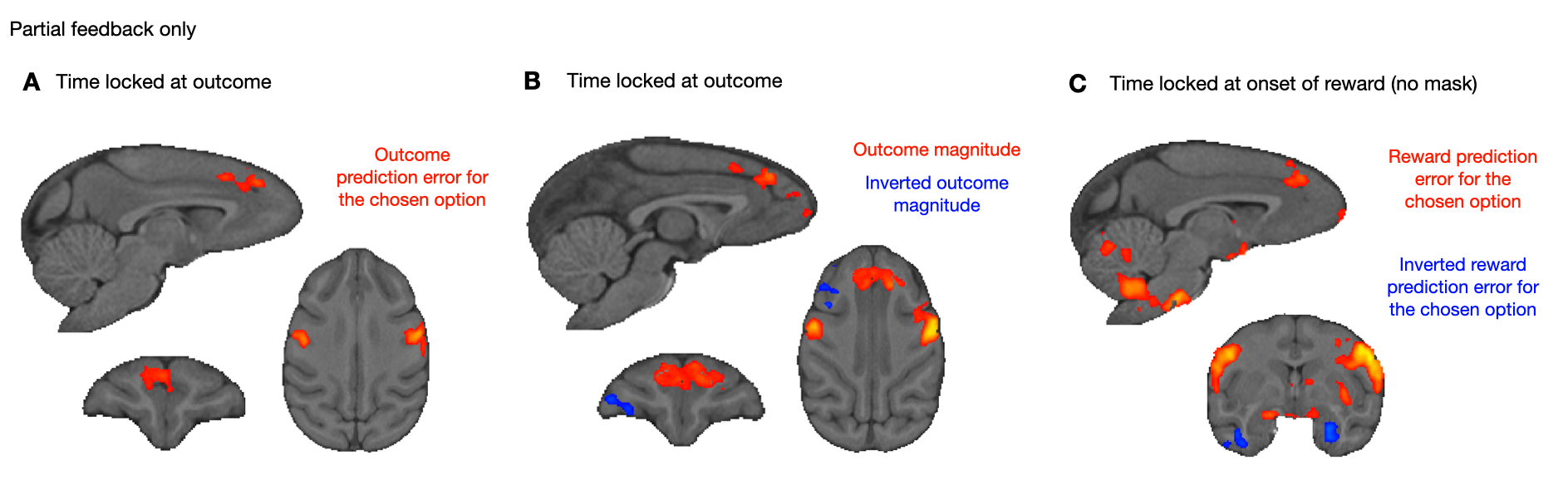

Supplement: S7 Fig — (A) In the partial feedback condition and at the time of outcome, we found 3 clusters of activity that were positively modulated by the chosen option prediction error in the medial prefrontal cortex and bilaterally in the somatosensory and motor cortex in our VOI (Cluster p < 0.05, cluster forming threshold of z > 2.3). (B) We found the same 3 clusters when we looked for a positive modulation by the magnitude of the chosen outcome. We additionally found 1 cluster of activity in the right lateral OFC that was negatively modulated by the magnitude of the chosen outcome. (C) When we time-locked our search to the onset of the reward (1 s after the display of the outcome, with a different GLM), we found the same clusters as in A, as well as the classic prediction error related activity in the ventral striatum and a negative prediction error in visual areas (see full map at https://doi.org/10.5281/zenodo.7464572) at the whole brain level. Data to reproduce the figure can be found at https://doi.org/10.5281/zenodo.7464572. GLM, general linear model; OFC, orbitofrontal cortex; VOI, volume of interest. (TIFF) [file pbio.3001985.s007.tiff]

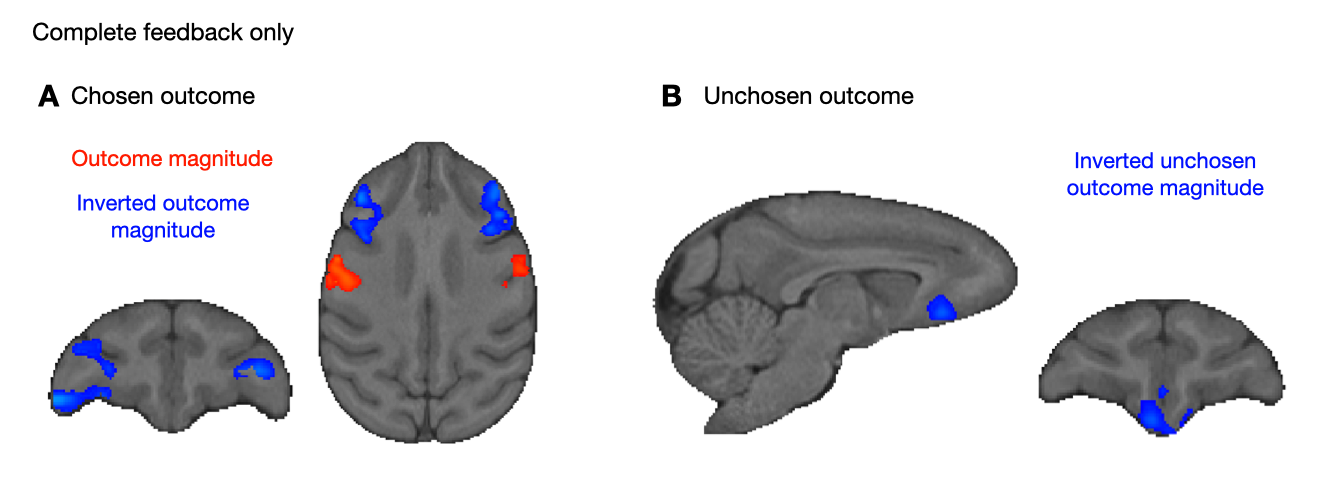

Supplement: S8 Fig — (A) In complete feedback sessions only, we found clusters for inverted chosen outcome magnitude activity in the right lOFC (47/12o) and bilaterally in the vlPFC and 2 clusters in the somatosensory/motor cortex [3]. (B) We found a cluster of activity for the inverted unchosen outcome magnitude in the cOFC and mOFC and the vlPFC. Data to reproduce the figure can be found at https://doi.org/10.5281/zenodo.7464572. cOFC, central orbitofrontal cortex; lOFC, lateral orbitofrontal cortex; mOFC, medial orbitofrontal cortex; vlPFC, ventrolateral prefrontal cortex. (TIFF) [file pbio.3001985.s008.tiff]

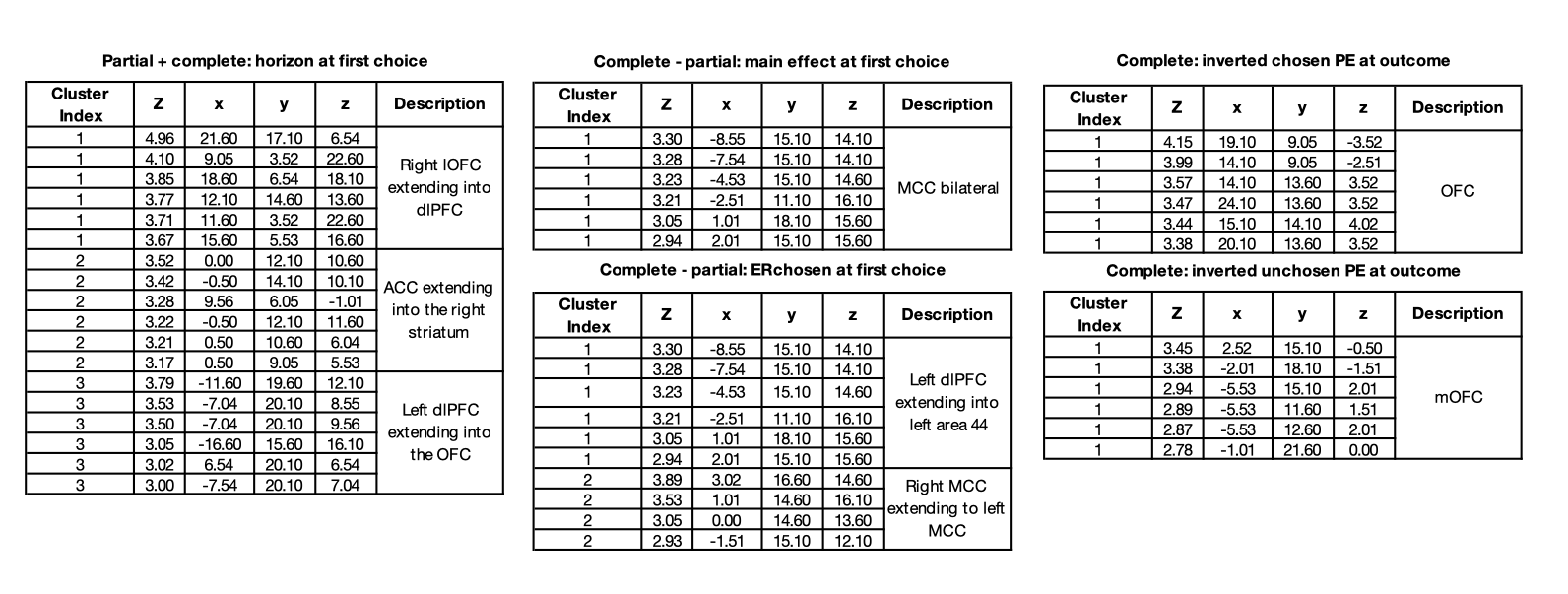

Supplement: S1 Table — Coordinates are given in the F99 standard space. (TIFF) [file pbio.3001985.s009.tiff]
