# Supplementary material for: Neural responses in macaque prefrontal cortex are linked to strategic exploration
Source: PLoS Biol. 2023 Jan 30;21(1):e3001985. doi: 10.1371/journal.pbio.3001985 (PMC9910800; doi:10.1371/journal.pbio.3001985)
Supplement: Attachment — Submitted filename: Response_to_reviewers_final.docx [file pbio.3001985.s010.docx]

Dear Dr. Dickson and reviewers,

Firstly, we would like to thank the reviewers for their time and efforts. We are grateful for their comments and constructive criticisms of our work. Throughout we have done our best to address their comments, highlighting textual changes in the manuscript in red and our replies here in blue.

Reviewer #1: In this new interesting work, Jahn and colleagues studied the behavioral and neural correlates of strategic decision-making in macaques. Through an innovative adaptation of the horizon task, previously used in human research by another group of researchers, the authors were able to study how monkeys strategically made different, well-defined, choices constrained by short vs. long decision horizon manipulations. Then, the authors used this task to examine the neural (fMRI) correlates of these behaviors.

While behavioral and neural bases of exploration vs. exploitation strategic decisions in rhesus macaques have been studied frequently through multiple approaches in the past, the use of the horizon task provided a novel and unique context to examine these strategic behaviors. In particular, this study could rely on the 'functional utility' of exploration decisions in a more constrained manner than some of the other existing studies due to the need to continue choosing sequentially in the long horizon condition. Furthermore, the use of fMRI allowed more holistic perspectives on how different decision variables are represented in this task.

Overall, the use of a novel task design combined with the careful analyses of decision variables at both behavioral and neural levels makes this research influential for future research in the domain of strategic decision-making. The behavioral and neural results are well-grounded in the model used and reveal an important change in expected value representation to promote exploration when there is a strategic benefit for doing so.

I believe this is a strong study with very interesting results. I did not find any major issues with this work but have some (mostly minor) comments that could further improve the manuscript and enhance its broader impact.

We are very happy to hear the reviewer found our work novel and interesting and did our best to address their comments.

1. A rather big portion of the Introduction is taken up by describing the task logic, methods, and results of the current work. Much of this information is again repeated at the beginning of the Results section. I suggest the authors cover more literature background and research motivation in the Introduction beyond introducing the horizon task and its relation to what the authors did in this manuscript. Doing so will more strongly position this work in light of the big-picture importance of studying strategic decision-making, in the brain and help readers, in addition to the Discussion section, to understand why this work is aimed at providing novel insights into strategic decision-making than other existing studies.

We thank the reviewer for this advice. We have revised our introduction in two ways. First, we now introduce previous studies of exploration in monkeys and humans and clearly explain how our work is complementary to what we already know. Secondly, we have shortened the presentation of the task and moved some details to the Result part.

In many species, most behaviors, including foraging, can be accounted for by simple behaviors – approach/avoidance of an observed and immediately available source of food – that require no mental representations. Exploration is by definition a non-value maximizing strategy (1,2), so in those models, exploration is often reduced to a random process, where noise in behavior can lead animals to change behavior by chance (1,3–6). However, in species relying upon spatially and temporally scattered resources, such as fruits, exploration is thought to be aimed at gathering information about the environment in order to form a mental representation of the world. Work in monkeys and humans has shown that primates are sensitive to the novelty of an option when deciding to explore (7–9). They sample novel options until they have formed a representation of their relative values compared to the available options. This work clearly showed that monkeys have a representation of the uncertainty and actively explore to reduce it. Similar results had been shown in humans, whose exploration is driven by the uncertainty about the options (10,11). However, it is still unknown whether monkeys have a specific representation of potential future action and outcomes that enables them to organize their behavior over longer time or spatial scale. We design a novel paradigm in monkeys – based on work in humans – to assess whether on how monkeys engage in strategic exploration, that is exploring only when it is useful for the future. Strategic exploration enables an animal to adapt to a specific context and is essential to maximize rewards over a longer time and spatial scale. For frugivorous animals such as primates, it might be critical for survival.

As in Wilson and colleagues’ original study, we manipulated whether the information could be used for future choices by changing the choice horizon (12). By comparing exploration in both conditions, we could test whether the animals reduced their reliance on value estimates (*random* exploration) and increased their preference for more uncertain options (*directed* exploration) when gathering information was useful for future choices in the long horizon (12). In addition, we manipulated the contingency between the choice and the information by varying the type of feedback that monkeys received. In the complete feedback condition, information was freely available, and we could probe whether monkeys decreased their exploration compared to the classic partial feedback condition. In humans, providing the complete feedback decreases decision noise (19) and improves learning (20–23) both of which are consistent with reduced exploration. A strategic explorer would only actively explore – and forgo immediate rewards – when it is useful for the future (long horizon) and that it is the only way to obtain information (partial feedback). In addition to behavioral data, neural data were collected using fMRI to probe the neural substrates of strategic exploration. Our analysis was focused on regions previously identified in fMRI studies on reward valuation and cognitive control in monkeys (24–29). Finally, we took advantage of the different feedback conditions to explore how monkeys update their expectations based on new information. Specifically, we investigated the behavioral and neural consequences of feedback about the outcome of their choice and – in the complete feedback condition – on counterfactual feedback from the alternative.

2. Strategic decisions frequently have idiosyncratic components as different monkeys may employ slightly different strategies for solving the same task even when their overall strategies might converge on average. It might be thus worthwhile to examine if there were individual differences in strategies over the course of a session or the course of the entire data collection (e.g., were these decision variables stationary or non-stationary?). Given that there is evidence of individual differences in Supp Figs 1-3, it would be informative to go deeper into how such individual differences might be reflected potentially differently across ACC/MCC, dlPFC, and OFC BOLD activations with respect to their functional roles suggested in this manuscript.

The reviewer asked several interesting questions that we will address in order. First, the reviewer asks if the decision variables (in our case the regression coefficients) are stable over the course of a session (1.a, interaction with trial number) and over the course of the data collection (1.b, interaction with session number). Second, the reviewer asks us to examine if the variability in the behavior (or across individuals) is reflected in BOLD activations (2).

In short, we found that the decision variables were stable over time and over the course of the session - or in any case, we found no linear nor clear trend for the regressors over time and we found no clear additional relationship between the brain and behavior (i.e., inter-session correlations) than the one we described in Fig 5B. Although the question of the inter-individual differences is extremely interesting, our dataset is not well suited to answer this question directly, either because the strategies that our three monkeys used were too similar and/or because we lack the statistical power to address it with an N=3.

For 1.a, we ran a logistic regression on the first choice and added the interaction of our regressor with the trial number. We used the exact same regressors as in Fig 2D and S1 and we z-scored all regressors for each session. Because we now fit each session separately, we had to remove two complete feedback sessions as there was not enough variance to fit all the regressors. The mean of the regression coefficients and the SEM (partial feedback condition N = 41, complete feedback condition, N=37) are plotted below. There was no interaction between the trial number and these parameters. None of the effects are significant, except for the effect of trial number on the repetition bias in the partial feedback condition (t(40)=2.0704, p=0.0449) but it is not significantly different than in the complete feedback condition (p=0.14).

For 1.b, we followed the same type of procedure but without the interaction with the trial number. Below we show the regression coefficients of the logistic regression on the first choice for each monkey and in the order of the session recording. We used the exact same regressors as in Fig 2D and S1 and we z-scored all regressors for each session. Because we now fit each session separately, we had removed one complete feedback session as there was not enough variance to fit all the regressors.

None of the effects described are consistent across monkeys and we did not detect any pattern that was significant. Overall, analyses 1.a and 1.b did not reveal a strong effect of the trial number nor the session number on the model parameters. Although the question of how strategic exploration emerges during learning is extremely interesting, our dataset does not seem well suited to address this question. Our monkeys were overtrained on this task and their decision variables were overall stable throughout the recording.

For 2, we would like to first highlight that although there are some individual differences in the magnitude of the decision variables used by our monkeys, their behaviors are remarkably consistent. Indeed, all monkeys showed a positive interaction between the feedback type and the horizon (Fig S2C) and all monkeys displayed some degree of counterfactual updating (Fig S5A).

The only inter-individual difference reported in the manuscript is Fig 5B because we hypothesized that the counterfactual prediction error for each session reflected how much they were able to learn from the counterfactual feedback, since this was a particularly hard feature of our task. Based on the reviewer’s comment we ran the additional correlations presented below. To do so, we ran a logistic regression at the session level and correlated the T-statistics of the regressor of interest with the T-statistic of the activation in the ROI of interest.

Below are some additional correlation analyses. For instance, we took the difference between the main activity during the first choice in the long horizon trials and in short horizon trials and the behavioral effect of the horizon of the sensitivity to expected value (pink = complete feedback, blue = partial feedback, purple = both). We found no correlation in the pgACC (top), the dlPFC (middle) nor the lOFC (bottom).

We also tried to correlate the main effect of the stimuli onset on the first choice between complete and partial feedback with the sensitivity to the expected value. We observed a borderline non-significant relationship (p=0.0525, positive relationship, one-tail T-test) in the MCC (top). Finally, when considering activations related to the chosen expected value, we found no correlation with the behavioral sensitivity to the expected value neither in MCC (middle) nor the dlPFC (bottom).

3. In P. 9, "However, we found no statistically reliable difference in the sensitivity to the uncertainty across the experimental conditions. Therefore, uncertainty did not play a key role in strategic exploration in our task." I am a bit surprised that there were no clear effects of uncertainty in monkeys' behaviors, as reducing uncertainty should be a major functional driver of decisions that are central to exploration. Unless I missed it, I don't see a place where the authors examined the effects of uncertainty at the neural activation level and how it might have influenced the neural representation of strategic decision variables in the brain regions examined. It would be surprising if uncertainty (variance) played no role in ACC/MCC, dlPFC, or OFC activations on signaling expected value or chosen/unchosen outcomes. If there are any effects, these modulations might further provide neural mechanistic insights into the computations performed by these brain regions for explore vs. exploit decisions. Either way, I suggest the authors discuss in some detail the effects of uncertainty (behavioral and neural) in the Discussion in relation to the main findings.

Given past studies emphasis on uncertainty-based exploration, our lack of clear uncertainty effects is indeed surprising. However, while we tested for the expected uncertainty preference in our data, there were several potential reasons for its absence.

Overall, animals looked like they were uncertainty averse. This surprising feature is likely explained by the fact that, particularly in the partial feedback condition, there is a strong correlation between number of times a side was picked and a reduction in uncertainty and macaques are known to be sticky. In other words, if the animals repeated choices, they will appear quite uncertainty averse, while they might just be sticky. While this design feature explains our unusual uncertainty main effects, it does not completely explain why there was no strong change in uncertainty aversion as a function of our manipulation (i.e., horizon and partial feedback). However, we speculate this might have to do with the fact that our uncertainty manipulation that went above number of samples, was so small that the animals might have favored other, simpler exploration strategies. In fact, our data strongly supports exploration by reducing the drive to pick the option of higher expected value as both options are often relatively similarly uncertain (at least when controlling for number of samples). Altogether, it seems that our subjects picked the non-default/nonpreferred/lower value option when it made sense to do so based on the context (horizon and feedback type) regardless of the precise distribution of outcomes (potentially because those were around a relatively tight mean to encourage learning) but biased towards preferring more reward samples that mimics risk aversion. We have added the following paragraph in the discussion:

Our results can also be compared to recent work in monkeys showing that monkeys choose novel options until they have an estimate of its value compare to other options (7–9), which can be interpreted as exploration for uncertainty reduction. Here, we found that monkeys did not seek to reduce overall uncertainty. This perhaps is because the number of reward samples was negatively correlated with uncertainty, and thus no option was novel in the sense that there was no information about it. Uncertainty-relate behavior was not modulated by the feedback type nor the horizon, hinting that uncertainty (rather than novelty) was not a driver of strategic exploration. This suggests species-specificity in exploratory strategy. In the future, a variation of our task could be used to test the effect of novelty on strategic exploration by offering zero information about one option.

Regarding neural correlates of uncertainty, we had a somewhat similar problem of not having huge range, but also the additional problem of interpretability, knowing that the animals likely didn’t track uncertainty very vigilantly. However, if we nonetheless look at uncertainty systematically in the brain, we see the following:

We systematically examined the following contrasts (and their inverse):

- partial and complete feedback: chosen uncertainty in long horizon vs. short horizon
- partial and complete feedback: chosen - unchosen uncertainty in long horizon vs. short horizon
- partial feedback: chosen uncertainty in long horizon vs. short horizon
- partial feedback: chosen - unchosen uncertainty in long horizon vs. short horizon
- partial vs. complete feedback: chosen uncertainty
- partial vs. complete feedback: chosen uncertainty in long horizon vs. short horizon
- partial vs complete feedback: chosen - unchosen uncertainty in long horizon vs. short horizon

We only found 1 cluster of activity for the following contrast: partial vs. complete feedback: chosen uncertainty, which showed more activity related to the uncertainty in the partial feedback in a cluster in the right somato-sensory cortex 1-2 and extending in the claustrum).

Given the lack of behavioral effect and the location of this activation, we find it very hard to interpret this result, so we chose not to include it in our manuscript.

Next, we examined the effect of uncertainty on the activations related to the main effect of horizon, feedback type and the expected value (Fig 4). We used the same methodology described in the response to comment #2.

We observed no correlation between the modulation of the sensitivity to the uncertainty by the horizon and the differential of activation during the long and the short horizon on the first choice (two-sided T-test). Note that the non-significant relationship in the pgACC is the opposite of what we would predict. A positive correlation means that a stronger activation in the long horizon corresponds to more uncertainty seeking in the short horizon, which is the opposite of what directed exploration would predict. There is some hint of this relationship in the partial condition in the dlPFC but the relationship is not significant.

We also found no relationship between the activation related to the feedback type nor the chosen expected value and the sensitivity to the uncertainty.

Despite the somewhat weak effects of uncertainty, it would not be fair to say that monkeys did not track the uncertainty at all as i) it affected their choices, as they preferred options with more pieces of information (Fig S1) *and* when the information was equal, options with the higher uncertainty (Fig S2), ii) we found a neural correlate of uncertainty in the medial prefrontal cortex extending into the frontal pole (from right 24c, 9m and 10mr bilaterally). We did not originally include this result as the main interest of our study was to look at how this effect was modulated by the horizon and the feedback. We have added a panel in Fig S6B as well as a reference to this finding in the Result section.

We also found a clear representation of the uncertainty about the chosen option on the first choice (when the magnitude of the uncertainty about the chosen option is equivalent in the partial and complete feedback conditions as no counterfactual feedback has yet been provided) in the right medial prefrontal cortex (24c and 9m) that extended bilaterally in the frontal pole (10mr) (Fig S6B).

**Fig. S6.** […] (**B**) In the partial and complete feedback conditions in our VOI and when taking the activity before the first choice only, we found 1 cluster of activity related to the inverse of the magnitude of the uncertainty about the chosen option in the right medial prefrontal cortex (24c and 9m) that extended bilaterally in the frontal pole (10mr) (Cluster *p* < 0.05, cluster forming threshold of z > 2.3).

However, throughout the manuscript we were careful not to over-interpret any of our findings related to the effect on baseline uncertainty on choices. That is, our design was set up to test how uncertainty is utilized differently based on the contextual factors of feedback type and horizon, but we did not intend to examine the monkey’s overall response to uncertainty. That being said, we show in the manuscript that while monkeys are overall averse to uncertainty (Fig S1), they are uncertainty-seeking if the initial pieces of information are matched (two on each side; Fig S2)

4. Throughout the manuscript, while the innovative and purposeful use of the horizon task was well discussed, the big-picture importance of having the functional utility of exploring vs. exploiting in the horizon condition compared to other existing studies of explore vs. exploit decision paradigms (e.g., two-armed bandit with fixed probability or with dynamic reward schedule) was not communicated as effectively as possible. Perhaps the authors should make this point clearly so that the readers can better grasp why this particular study was able to demonstrate certain aspects of behavioral and neural strategic decision-making in monkeys compared to other existing work.

We have rewritten the introduction and we hope that the relationship of this task and previous exploration tasks is now clearer to the reader (same insert as in the answer to comment #1).

In many species, most behaviors, including foraging, can be accounted for by simple behaviors – approach/avoidance of an observed and immediately available source of food – that require no mental representations. Exploration is by definition a non-value maximizing strategy (1,2), so in those models, exploration is often reduced to a random process, where noise in behavior can lead animals to change behavior by chance (1,3–6). However, in species relying upon spatially and temporally scattered resources, such as fruits, exploration is thought to be aimed at gathering information about the environment in order to form a mental representation of the world. Work in monkeys and humans has shown that primates are sensitive to the novelty of an option when deciding to explore (7–9). They sample novel options until they have formed a representation of their relative values compared to the available options. This work clearly showed that monkeys have a representation of the uncertainty and actively explore to reduce it. Similar results had been shown in humans, whose exploration is driven by the uncertainty about the options (10,11). However, it is still unknown whether monkeys have a specific representation of potential future action and outcomes that enables them to organize their behavior over longer time or spatial scale. We design a novel paradigm in monkeys – based on work in humans – to assess whether on how monkeys engage in strategic exploration, that is exploring only when it is useful for the future. Strategic exploration enables an animal to adapt to a specific context and is essential to maximize rewards over a longer time and spatial scale. For frugivorous animals such as primates, it might be critical for survival.

5. Differentiated or common roles of different brain regions tracking decision variables were interesting. Were there any different functional connectivity patterns among different brain regions for exploration vs. exploitation and in long vs. short horizons? Such network-level analyses may provide additional support on how different brain regions work together to compute strategic choices.

We unfortunately never tried full whole-brain network-level analyses as the non-human primate MRI pipeline we used did easily allow for such analyses without us building up the required pipeline ourselves. We did, however, examine connectivity patterns between our ROIs using PPIs. Unfortunately, our results remained mostly inconclusive and were hard to interpret so we did not include any of them in the main manuscript. Having said this, we report some of our tentative analyses below for your interest.

We examined whether the connectivity between our frontal PE ROIs (defined at outcome) and the cingulate ROI encoding expected value (defined at choice) changed as a function of PEs. Specifically, here we are prediction activity in the area 24 ROI from the area 13 ROI. We included regressors for the BOLD signal in area 13, the main effect of both chosen and unchosen PEs, and the interaction of the BOLD timecourse with both types of PE. Additionally, we also included the average BOLD signal within our frontal mask. Below we plot the interaction of the BOLD signal in 13 with chosen and unchosen PE. As can be seen, there is a tendency for the connectivity to increase with chosen PE and decrease with unchosen PE.

However, when repeating the same analysis for the ROI we defined in the neighboring region 14, this weak effect disappears:

Both the ROIs for areas 13 and 14 were defined based on the unchosen PE. When we repeat the same analysis for area 12o, in which we found only chosen but no unchosen PE activity, we also see a similar pattern of connectivity:

We found similarly inconclusive patterns for connectivity between the other ROIs and effects discussed in the manuscript. As such, we decided not to include any of these analyses in the main text or the supplements.

6. Fig. 1B, in the distributions of Expected Value and Uncertainty, there are some thin lines that should be deleted (second and third column of the distribution graphs) - I believe these are remainders left over while making the Fig.

We apologize for the confusing aspect of Fig 1B, the light marks represent the previous trial estimates. Although it is indicated in the Fig caption “Thick lines correspond to post outcome estimate and thin lines to pre-outcome estimates (from the previous trial).”, we agree that it is confusing for the reader. We have added arrows and the caption “Update” on the Fig to make this clearer.

7. P. 11, the results section at the top of this page has some sentences that should refer to Fig. 3E, rather than Fig. 3D.

We thank the reviewer for noticing this mistake. We have corrected all the wrong references to Fig 3D.

Reviewer #2: Jahn CI et al., Strategic exploration in the macaque's prefrontal cortex

The authors describe data from a study in which they trained monkeys on a primate variation of the horizon's task, to study the explore-exploit tradeoff, and carried out fMRI while animals executed the task. They found that animals relied more on the expected value in short horizon than long horizon choices. There choices did not, however, depend on the uncertainty of the options. Comparisons of different conditions showed that there was increased activation in areas 24, 46 and 47/12 for long-horizon vs. short-horizon trials, and increased activation in area 46 for partial vs. complete feedback.

This manuscript addresses important questions about the behavioral and neural mechanisms that underlie exploration vs. exploitation. The behavioral paradigm is well-controlled and is based on the Horizon's task developed previously by Robert Wilson. This task allows for explicitly testing specific hypotheses about the factors that underlie exploratory behavior. Overall, this is an interesting manuscript. I do have several comments which should be addressed.

We are glad that the reviewer liked the manuscript and would like to thank the reviewer for their positive and critical examination of our manuscript.

Comments

1. The abstract and introduction conflate foraging and the explore-exploit tradeoff. Foraging and the explore-exploit tradeoff, however, refer to different behavioral/theoretical processes. Stephens and Krebs (reference 1) developed the marginal value theorem to account for foraging. Within this framework, the choice to leave a patch results in a random sample from a known distribution of other patches. Thus, there is no learning within the theoretical framework that has been developed to describe foraging. The explore-exploit tradeoff on the other hand comes from the reinforcement learning literature and refers specifically to the process being studied in this manuscript. In the explore-exploit tradeoff the goal is specifically to learn, or gather more information, about some probability distribution. While there is of course an intuitive or folk similarity in the terms exploration and forage, they do not refer to the same phenomenon as studied in the literature. If you would like to stick with the term foraging, I would suggest stating that you are not using it in the way in which it was originally defined. I would, however, suggest removing reference to foraging.

We agree with the reviewer that the optimal foraging theory is theoretically different from the exploration we study, but we do believe it is crucial to contextualize our results in an ecological perspective. In the introduction and abstract, we chose to use the term foraging in the literal sense of gathering resources rather than refer to optimal foraging theory exclusively. Primates have especially large brains for their body weight, which are energy greedy. It is believed that many higher executive functions have evolved to solve the problem of gathering enough food which often happens in the context of uncertainty and learning. In particular, macaques eat fruits that are seasonal and require a knowledge of the environment and - as is at the heart of our paradigm - strategic exploration. We have edited the first sentence of the introduction so as not to conflate the optimal foraging theorem and exploration. We have also made explicit the link between foraging and learning as the reviewer suggested.

In many species, most behaviors, including foraging, can be accounted for by simple behaviors – approach/avoidance of an observed and immediately available source of food – that require no mental representations. Exploration is by definition a non-value maximizing strategy (1,2), so in those models, exploration is often reduced to a random process, where noise in behavior can lead animals to change behavior by chance (1,3–6). However, in species relying upon spatially and temporally scattered resources, such as fruits, exploration is thought to be aimed at gathering information about the environment in order to form a mental representation of the world.

2. A number of papers, some of them recent, should be cited. The work of Vincent Costa in monkeys and recently in humans (Hogeveen J et al., Neuron, 2022; Costa VD and Averbeck, J Neurosci, 2020; Costa VD et al., Neuron, 2019). Work by Becket Ebitz (Ebitz RB et al., Neuron, 2018), and work by Michael Frank (Badre D et al., Neuron, 2012; Cavanagh JF et al., Cer Cortex, 2012), for example.

We agree with the reviewer that our introduction and literature review was too bare bones previously. We now introduce the work mentioned by the reviewer, as they are relevant and timely, in the introduction. In the discussion, we have also added a mention of the difference in the type of uncertainty that is modulated in our and their tasks and why these differences are interesting and important.

Introduction

Work in monkeys and humans has shown that primates are sensitive to the novelty of an option when deciding to explore (7–9). They sample novel options until they have formed a representation of their relative values compared to the available options. This work clearly showed that monkeys have a representation of the uncertainty and actively explore to reduce it. Similar results had been shown in humans, whose exploration is driven by the uncertainty about the options (10,11). However, it is still unknown whether monkeys have a specific representation of potential future action and outcomes that enables them to organize the behavior over longer time or spatial scale. We design a novel paradigm in monkeys – based on work in humans – to assess whether on how monkeys engage in strategic exploration, that is exploring only when it is useful for the future. Strategic exploration enables an animal to adapt to the specific context and is essential to maximize rewards over a longer time and spatial scale. For frugivorous animals such as primates, it might be critical for survival.

Discussion

Our results can also be compared to recent work in monkeys showing that monkeys choose novel options until they have an estimate of its value compare to other options (7–9), which can be interpreted as exploration for uncertainty reduction. Here, we found that monkeys did not seek to reduce overall uncertainty. This perhaps is because the number of reward samples was negatively correlated with uncertainty, and thus no option was novel in the sense that there was no information about it. Uncertainty-relate behavior was not modulated by the feedback type nor the horizon, hinting that uncertainty (rather than novelty) was not a driver of strategic exploration. This suggests species-specificity in exploratory strategy. In the future, a variation of our task could be used to test the effect of novelty on strategic exploration by offering zero information about one option.

3. It would be useful to report ANOVA results for the data shown in Fig. 2B. A direct examination of main and interaction effects of feedback and horizon would be useful. Means from each session could be entered into the analysis. It's also somewhat surprising that the regression weight for expected value is higher in the complete feedback condition (Fig. 2D) but the probability of choosing the highest EV option is not higher in 2B. Perhaps some of the other variables from the logistic regression are driving this. Are there strong correlations, for example, between repetition bias and EV?

In brief, in Fig 2B, we show the raw probability to choose the option with the highest expected value to i) give a sense of the general scale of value preference in percentages (rather than regression weights) and ii) show that the monkeys generally perform the task above chance level. However, using the raw accuracy is not a suitable test for the manipulation because other factors make this percentage a very noisy estimate. In other words, while the interaction between the horizon and the feedback type is the same as in Fig 2D, the variance of the estimate is a lot larger because it doesn’t control for the fact that in some sessions, animals are less accurate because of a side bias working against them (see full explanation below). Therefore, we only do such statistics on the expected value effect because that is a better, quantitative version of accuracy as defined as the drive to pick highest expected value option.

One very important confound is an inherent side-bias the monkeys exhibit during each session. This side-bias is very likely due to task-irrelevant factors: while doing the task, the monkeys were seated in the sphinx position in a purpose build chair, and during each session they had adjusted their bodies slightly to put a bit more weight one of their shoulders, which makes it easier to respond with one hand rather than the other. It is important to control for such a side-bias in our analysis to obtain better behavioral measures. This is not done for the data as shown in Fig 2B, but it is done for the full model fit shown in Fig 2D (the intercept of the regression model regresses out the side-bias and repetition bias). Finally, the raw accuracy is blind to the magnitude of the difference in expected value between the two options. It does not distinguish between not choosing the best option when the values are very close and when they are further apart. Thus, doing the statistics for the model fit shown in Fig 2D allows us to account for confounds such as side bias and repetition bias, and to clearly isolate the effect of expected value on choice. In the study that inspired our task design, Wilson et al. (2014) also do not report any statistics on the raw accuracy measures but fit a model to their data that, among other variables, also controls for a side-bias.

Below we show the regression coefficients of the logistic regression on the first choice. We used the exact same regressors as in Fig 2D and S1 and we z-scored all regressors for each session. We removed one complete feedback session as the iteration limit was reached when fitting the linear regression model.

We did not find any correlation between the sensitivity to the expected value and the repetition bias.

Partial feedback condition: r = -0.26, p = 0.1, N = 41

Complete feedback condition: r = -0.15, p = 0.33, N = 40

All sessions: r = -0.17, p = 0.12, N = 81

We did not find any correlation between the interaction between the horizon length and the sensitivity to the expected value and the repetition bias.

Partial feedback condition: r = -0.23, p = 0.14, N = 41

Complete feedback condition: r = 0.06, p = 0.70, N = 40

All sessions: r = -0.06, p = 0.61, N = 81

We are therefore confident that our model allows us to isolate the specific effect of expected value on choices, independently of other confounds.

4. In the Horizon's task, exploration is divided into directed and undirected (or random) exploration. In the current manuscript, the term strategic exploration is used. However, there are no effects of uncertainty on exploration, and therefore monkeys are only using undirected exploration. Is random exploration strategic? Is the strategic referring to the increase in exploration in the partial feedback and long horizon in the partial condition? Do these effects come out of the ANOVA (comment 3?). It would be useful to discuss explicitly, directed and undirect exploration, and how these relate to the current results. Avoiding uncertainty, which the animals show, is difficult to fit within this framework. Perhaps some mention of this would be useful.

We agree with the reviewer and we now realize that our description of our model in the results and discussion sections was lacking in several ways. Our results suggest that the monkeys do not preferentially explore the option with higher uncertainty which has previously been used to define random and directed exploration. However, as the animals adjust their degree of exploring the generally non-preferred option based on value and context, we also don’t know whether such exploration is strictly speaking random as they increase such exploratory behavior as a function of feedback type and horizon length, potentially as a result of a targeted strategy to learn about its mean (see our response to reviewer 1 about potential reasons of a lack of uncertainty modulation). However, it is of course true that according to the common definition of directed vs random exploration, what we call ‘strategic exploration’ is an adjustment of random exploration depending on both the feedback type and horizon. We have done so in several ways: we now explicitly refer to random and directed exploration in the introduction, results and discussion and we have added a supplementary figure (Fig S3) that directly shows our results in this frame.

Introduction

We found that rhesus monkeys engaged in strategic exploration by decreasing their reliance on expected values (*random* exploration) when it was useful for the future (long horizon) and that active sampling was the only way to obtain information (partial feedback). Neurally, we found prefrontal strategic exploration signals in the ACC/MCC and dlPFC. However, we did not find a significant effect modulation by the horizon and feedback type of the effect of uncertainty (*directed* exploration) on choices.

Results

In other words, animals strategically modulated the degree to which they used *random* exploration both depending on the horizon length and feedback type (Fig S3A).

This indicates that our macaques did not use *directed* exploration to strategically guide their choices (Fig S3).

Discussion

However, contrary to human behavior, our monkeys only adapted by reducing their reliance on expected value (i.e., exploitative value) on choices, which corresponds to the degree to which they use *random* exploration. Humans also increase their preference for the most uncertain option when exploration is useful for the future, which has been referred to as *directed* exploration (12).

**Fig. S3.** Equivalency between our linear regression and the framing in terms of *random* and *directed* exploration. (**A**) With the uncertainty regressor. We find that monkeys modulate their sensitivity to the expected value depending on the horizon and the feedback type, which is equivalent to the *random* exploration parameter, the softmax noise, which is the inverse of the expected value regressor. However, we find no modulation of the uncertainty by the horizon nor the feedback type, which is equivalent to the *directed* exploration parameter, the uncertainty bonus, which is the uncertainty regressor divided by the expected value regressor. (**B**) Same as A but with the number of available information rather than the uncertainty. Error bars indicate standard deviation.

We have added the following clarification in the manuscript and discussion of the effect of uncertainty on choices (same insert as in the answer to comment #2).

Our results can also be compared to recent work in monkeys showing that monkeys choose novel options until they have an estimate of its value compare to other options (7–9), which can be interpreted as exploration for uncertainty reduction. Here, we found that monkeys did not seek to reduce overall uncertainty. This perhaps is because the number of reward samples was negatively correlated with uncertainty, and thus no option was novel in the sense that there was no information about it. Uncertainty-relate behavior was not modulated by the feedback type nor the horizon, hinting that uncertainty (rather than novelty) was not a driver of strategic exploration. This suggests species-specificity in exploratory strategy. In the future, a variation of our task could be used to test the effect of novelty on strategic exploration by offering zero information about one option.

5. The figure legends state that error bars are S.E.M. but they do not give the N.

We apologize for this oversight. We have corrected all figure captions to include the N for the SEM. Below we report the N but not the full figure caption for space consideration.

Fig 2 B: Mean across sessions (partial feedback: 41 sessions, complete feedback: 40 sessions).

Fig 2 C: Mean across all sessions (81 sessions).

Fig 3 A: Mean across sessions (partial feedback: 41 sessions, complete feedback: 40 sessions).

Fig 3 B: Mean across all sessions (81 sessions).

Fig 3 C: Mean across complete feedback sessions (40 sessions).

Fig 3 D: Mean across sessions (partial feedback: 41 sessions, complete feedback: 40 sessions).

Fig 4 B: Mean across sessions (partial feedback: 40 sessions, complete feedback: 34 sessions).

Fig 4 D: Mean across sessions (partial feedback: 40 sessions, complete feedback: 34 sessions).

Fig 4 E: Mean across sessions (partial feedback: 40 sessions, complete feedback: 34 sessions).

Fig 4 C: Mean across complete feedback sessions (34 sessions).

Fig 4 D: Mean across complete feedback sessions (34 sessions).

6. F and t- stats and N are given for the imaging data, but not for the behavioral data. More detailed statistics should be given for the behavioral data.

We have added a more detailed description of Fig 2B that includes the T-statistics (changes in red).

For the two horizon lengths and in both feedback conditions, accuracy was above chance level (t-test compared against a mean of 0.5; partial feedback short horizon: t(40) = 10.0294, p<0.001, partial feedback long horizon: t(40) = 8.9300, p<0.001, complete feedback short horizon: t(39) = 7.9056, p<0.001, complete feedback long horizon: t(39) = 8.9634, p<0.001, Fig 2B). Therefore, monkeys used the information provided by the informative observations on each trial to guide their choices.

We have added the N for Fig 2C. This figure was intended for visual inspection of the ‘raw’ data. Here the proper statistical analysis would be a logistic regression of the effect of expected on choices which is reported in Fig 2D.

Monkeys also adjusted their choices to variations in expected value, as can be seen when pooling together both feedback conditions and horizon lengths (Fig 2C, see statistical significance in Fig 2D).

For Fig 3A, it would be very lengthy to report the t-statistics for each of the 8 conditions (feedback type x 4 choices) so we have simply added a sentence about the high significance of the results for all conditions. We have added the results of the T-statistic of the distribution linear regression coefficient of choice number onto the accuracy for each feedback type (changes in red).

On such trials, monkeys’ accuracy (defined as choosing the option with the highest expected value according to the model) was always above chance level (T-test compared against a mean of 0.5; all p<10-10) and increased as they progressed through the sequence (t-test compared against a mean of 0 of the distribution regression coefficients of the trial number onto the accuracy (both z-scored) for each session; partial feedback condition: t(40)=11.3653, p<0.001, complete feedback condition: T(39)=5.6590, p<0.0001) (Fig 3A).

As for Fig 2C, we have added the N in the figure caption of Fig 3B-C and the proper statistical analysis is reported in Fig 3E. We have added an explanation for looking at this metric and about the statistics:

We inferred that this improvement was due to the use of the information collected during the choices. To examine this, we isolated the change in expected value compared to the initial ‘observation phase’ (see Material and Methods). We found that monkeys were sensitive to the change in expected value both for the chosen option (in the partial and complete feedback conditions) and the unchosen option (counterfactual feedback in the complete feedback condition only) (Fig 3B-C, see statistical significance in Fig 3E).

In the previous version of the manuscript, we mistakenly referred to Fig 3B for the repetition bias, we have corrected this error and now refer to Fig 3D. We have moved the description of this effect after the description of the results of Fig 3B-C. As for Fig 3A, it would be very lengthy to report the t-statistics for each of the 8 conditions (feedback type x 4 choices) so we have simply added a sentence about the high significance of the results for all conditions against a distribution with a mean at 0.5. We have extended our description of the repetition bias, adding a comparison of first choice vs. the subsequent ones (again we have compressed the results to only the lower p-value) and the results of the T-statistic of the distribution linear regression coefficient of choice number (2 to 4, as there is a clear non-linearity from 1 to 4) onto the probability to repeat the same choice for each feedback type (changes in red).

Monkeys displayed a significant tendency to choose the same option (t-test compared against a mean of 0.5; all p<10-6), which sharply increased after the first trial (paired t-test between the first choice and the subsequent choices; all p<10-10) and kept increasing after the first choice (t-test compared against a mean of 0 of the distribution regression coefficients of the trial number onto the probability to choose the same option (both z-scored) for each session; partial feedback condition: t(40)=5.3026, p<0.001, complete feedback condition: t(39)=3.1265, p=0.0033) (Fig 3D).

Finally, we have added a sentence to describe how the model isolated that the difference between the partial and complete feedback condition can be attributed to the repetition bias rather than a bias for choosing the option most often chosen, which scales with the choice number.

Monkeys also preferred to choose the option most chosen (p<0.000001 in the partial condition and p<0.000001 in the complete; one-sided test), which explained the increase in repetition bias over time, but this was not affected by the feedback type (partial > complete: p=0.309) (Fig S5).

7. The Bayesian statistics are fine. But what if a t-test were used on the parameter comparisons for the logistic regression? Can you report t and p values for these comparisons? It's not clear what is gained by using the Bayesian framework in this situation.

Using STAN allows us to account for the variability in the identity of the subject (which is particularly useful here as we have 3 subjects who perform several sessions). For each regressor and each animal we estimated the Gaussian distribution that session-level regressors are most likely drawn from. We then obtained the group-level estimates of the coefficients by averaging across animals, which cannot be done using standard mixed-effects modeling. As such, the Bayesian framework we used allows us not only to estimate the full posterior distributions of our parameters (which are shown in the supplementary materials and might be of interest to some readers) but also account for animal-specific variance in a better way.

Below we show the regression coefficients of the logistic regression on the first choice. We used the exact same regressors as in Fig 2D and S1 and we z-scored all regressors for each session. We display the mean across sessions (partial feedback: 41 sessions, complete feedback: 39 sessions) and the error bars are the standard error to the mean. Because we are now doing an individual fit for each session rather than the hierarchical fit of all the data, we had to remove one complete feedback session as there was not enough variance to fit all the regressors.

In the partial feedback, we found a positive effect of the expected value on choices (t(40)=9.0367, p<0.001, one-sided-t-test) and positive interaction between the expected value and the horizon (t(40)=2.3233, p=0.0127, one-sided-t-test), replicating the results of Wilson et al. 2014.

In the complete feedback, we found a positive effect of the expected value on choices (t(38)=6.2844, p<0.001, one-sided-t-test) and no significant relationship between the expected value and the horizon (t(38)=-0.5875, p=0.5604, two-sided-t-test).

We found that the difference in the effect of expected value between the complete and the partial feedback conditions was positive but non-significant (t(78)=1.4746, p=0.0722, one-sided-T-test) when doing such frequentist statistics. However, the crucial interaction effect with the horizon was significant i.e. difference between horizons by partial/complete feedback condition (t(78)=1.6818, p=0.0483).

As can be seen, the effects are very similar to those described in the manuscript. In particular, the key manipulation: the interaction between the effect of horizon and feedback is significant. We used Bayesian statistics in order to get a more reliable estimate of the regression coefficient, giving us confidence in the results we show.

Reviewer #3: This manuscript describes an attempt to implement the horizon task pioneered by Wilson and colleagues (2013) in rhesus macaques. An operating assumption is that monkeys like humans would use directed exploration, guided by uncertainty, in deciding whether to sample one option over another. To test this assumption authors adapted the version of the task tested in humans. The monkeys first observed visual cue presentations that signaled information about the potential value in choosing either cue. Equal (2 observations of visual feedback about each cue) or unequal information (3 observations of visual feedback for one cue and 1 observation of the other cue) was provided about each of the two cues during this observation phase. No primary rewards were experienced during the observation phase. The monkeys were then visually signaled by the height of two gray vertical bars on the left and right side of the screen, that they would have either one (short horizon) or four (long horizon) opportunities to choose between the two options following the observation phase. After each choice, the monkeys received between 0 and 10 drops of juice determined by the length of visual feedback associated with their choice. The monkeys completed the task inside an MRI scanner to enable assessments of brain activity related to encoding of the choice horizon and expected value. One additional manipulation was that following their choices the monkeys were provided with either partial feedback (i.e. they only received visual feedback about the option they chose) or complete feedback (i.e. they were shown visual feedback about both the chosen and unchosen option, but only received primary reward related to the option they chose). The partial versus complete feedback manipulation occurred across the individual test sessions, whereas the horizon and information manipulations occurred within a session. The authors used a Bayesian model to quantify the expected value and uncertainty associated with choosing each option prior to each instance of visual feedback in the observation and choice phases of each trial. The authors focus on a reduced reliance on the expected value in deciding which option to choose as indicative of the monkeys' using "strategic exploration" to decide when to explore, predicting increased exploration in long versus short horizons when the monkeys received partial feedback. Whereas exploration should not differ between the horizon conditions when complete feedback is provided. Although the authors believe they have sufficient behavior and neural evidence to support this hypothesis, their claims are not well supported. I have serious doubts about whether the monkeys were even sensitive to the horizon manipulation and more fundamental behavioral analyses that hew closely to the data and match those performed previously in humans are needed before interpreting the reported fMRI contrasts or claiming to have identified differences in humans versus non-human primates use of strategic exploration. My major concerns are outlined below:

1. The authors need to provide more direct evidence, replicating analyses from Wilson et al. (2013) that demonstrates the monkeys were sensitive to the horizon manipulation. While their accuracy is above chance it is not apparent that the accuracy measures directly differ by the horizon or feedback conditions based on the error bars in the plot provided in Fig 2. The horizon task is set up to measure directed versus random exploration, explicitly, by assessing how either the selection of the more informative, uncertain option varies as a function of the difference in the expected value of the two options based on the observation phase when unequal information is provided (i.e. the number of forced choice trials in the observation) or preference for a particular choice option (i.e. right or left) varies with the difference in the expected value when equal information is provided. When a non-linear choice function (e.g. sigmoid) is fit to the behavior in this way the slope and intercept of the function can be used to quantify random exploration (slope) and how directed exploration is shaped by an information bonus. Given that the manuscript potentially describes the first implementation of the horizon task in nonhuman primates, these fundamental analyses that build a bridge to human studies and that do not rely on the implementation of a particular computational model must be added to the main body of the manuscript. Especially critical is fitting these functions to choices on the first trial in the partial feedback sessions when equal or unequal information is presented. But I suspect, given the result from the Bayesian model that the monkeys avoided uncertainty, that these analyses will reveal that the monkeys are not using directed exploration at all, that the information bonuses are equivalent across the two horizons, and that the documented "strategic exploration" is simply an increase in random exploration denoted by a smaller slope in the long vs. short horizon condition stemming from the animals being less exploitative. If this is the case then it casts the fMRI analyses in a very different light, especially the contrast of the activity as a function of horizon. If this contrast is effectively highlighting random rather than directed exploration this might be one reason that cingulate rather than the frontal pole of prefrontal cortex is activated differently under the two conditions. To summarize, the authors need to show, using some behavioral metric that doesn't rely on the Bayesian regression, using only the first choice trials where value and uncertainty are decoupled by the forced choice manipulation that convincingly shows the animals were sensitive to the horizon manipulation.

The reviewer is raising several points. 1) Are the animals sensitive to the horizon manipulation i.e., do they care about the horizon? 2) Are they sensitive to the interaction of horizon by feedback type, i.e., the effect we used to indicate strategic exploration and how is strategic exploration related to *random* and *directed* exploration? 3) Are our results identical to Wilson et al (2014) and if not in what way to they differ? Do they need to be identical to be meaningful?

In brief. 1) We can categorically state that we have evidence for the fact that animals changed their behavior based on the horizon manipulation. We have a main effect and an interaction effect with feedback type. 2) This interaction effect is tested by using a simple regression on the animals’ *first choices* in the sequence (for long horizon). Strategic exploration refers specifically to this interaction effect and we now state clearly that it applies to random exploration but not directed exploration. We have added an explanation for how our model is mathematically equivalent to the model in Wilson et al. 2014 (see more details further down). 3) Our results differ from Wilson in feature, i.e., uncertainty sensitivity (~ directed exploration). We talk about this also for R1 and R2. In short, we believe this is a genuine difference between the tasks but whether it is due to design differences or species differences in exploration we cannot definitively test. However, we do not believe that uncertainty modulated choices are the only way to show exploration strategies as the animals moderate their exploration quite clearly between sessions (partial vs complete feedback) as well as within a session (short vs long horizon).

To clarify further about our answer to the first point, Fig 2B is the “raw accuracy” i.e., the proportion of high value exploitative choices in our data, not accounting for any other behavioral measures. The reason we included this plot was to show that the monkeys learned to do the task and that their choices were above chance for all horizon types and feedback conditions, but we admit that its inclusion is in retrospect confusing. We do not perform any further statistics on this measure as we think it is fundamentally flawed for testing our key manipulations as it is not accounting for some other important aspects of how the monkeys behave during the task nor accounts for the nature of errors in a way a logistic regression does. In particular, one very important confound is an inherent side-bias the monkeys exhibit during each session. This side-bias is very likely due to task-irrelevant factors: while doing the task, the monkeys were seated in the sphinx position in a purpose build chair, and during each session they had adjusted their bodies slightly to put a bit more weight one of their shoulders, which makes it easier to respond with one hand rather than the other. It is important to control for such a side-bias in our analysis to obtain better behavioral measures. This is not done for the data as shown in Fig 2B, but it is done for the full model fit shown in Fig 2D (the intercept of the regression model regresses out the side-bias). Finally, the raw accuracy is blind to the magnitude of the difference in expected value between the two options. It does not distinguish between not choosing the best option when the values are very close and when they are further apart. This is why the pattern of the mean accuracies (partial + long horizon lowest) is identical to the pattern for the beta weights but the errorbars are comparatively very large as many confounding elements are not considered. Doing the statistics for the model fit shown in Fig 2D allows us to account for confounds such as side bias and repetition bias, and to clearly isolate the effect of expected value on choice. We furthermore note that the study that inspired our task design, Wilson et al. (2014) also do not report any statistics on the raw accuracy measures but fit a model to their data that, among other variables, also controls for a side-bias.

To elaborate further on the second point on the nature of the effect and whether it needs to be directed exploration as in Wilson et al. (2014), we now realize that our description of our model in the results and discussion sections was lacking in several ways. The reviewer rightfully points out that our results suggest that the monkeys do not use directed exploration but rather vary their level of random exploration depending on the feedback type and horizon length. We fully agree with this description of our results. What Wilson et al. (2014) called information bonus in their analysis was replaced by uncertainty in ours (we explain why further below), and when we highlighted that there was no effect of uncertainty by feedback type or horizon length, we meant this to suggest that our monkeys did not use directed exploration, but we see that this should have been spelled out more clearly. Thus, what we call ‘strategic exploration’ is an adjustment of random exploration depending on both the feedback type and horizon. We clarify the relationship between strategic, random and directed exploration in several ways: we now explicitly refer to random and directed exploration in the introduction, results and discussion and we have added a supplementary figure (Fig S3) that directly show our results in this frame.

We can easily interpret our results in the frame of random and directed exploration because of the mathematical equivalence in modelling compared to Wilson et al, we can show that the linear regression we use is equivalent to the choice model that Wilson and colleagues used in their 2014 paper (plus a few additional regressors we added). We start with the formula we use in the manuscript:

$$P\left( {right}_{t} \right)=\sigma\left( b_{SB}+b_{RB}{RB}_{t}+b_{horizon}{horizon}_{t}+b_{EV}{EV}_{t}+b_{U}U_{t}+b_{ERhorizon}{ER}_{t}{horizon}_{t}+b_{Uhorizon}U_{t}{horizon}_{t} \right)$$

For simplicity, let us delete all the regressors not used by Wilson and colleagues. That is, we only include a side bias, an information bonus, and the main effect of reward. The regression becomes

$$P\left( {right}_{t} \right)=\sigma\left( b_{SB}+b_{EV}{EV}_{t}+b_{U}U_{t} \right)$$

The regressors ${EV}_{t}$ and $U_{t}$ both denote the difference in expected reward and expected uncertainty on trial *t* between the right and the left option on screen. We drop the subscript *t* and make this explicit by writing

$$P_{right}=\sigma\left( b_{SB}+b_{EV}{(EV}_{\boldsymbol{right}}-{EV}_{\boldsymbol{left}})+b_{U}{(U}_{\boldsymbol{right}}-U_{\boldsymbol{left}}) \right)$$

What we call *EV* is called *R* by Wilson and colleagues, and instead of using uncertainty, Wilson and colleagues use an information bonus *I.* For a discussion of the difference between uncertainty and the information bonus, please refer to our earlier section in this response letter but we want to note here that no main results change depending on whether we include *U* or *I* as a regressor in our model. We now change our notation to match the notation of Wilson and colleagues, and explicitly write out the sigmoid function $\sigma(\cdot)$:

$$P_{right}=\frac{1}{1+exp\left( b_{SB}+b_{EV}{(R}_{\boldsymbol{right}}-R_{\boldsymbol{left}})+b_{U}{(I}_{\boldsymbol{right}}-I_{\boldsymbol{left}}) \right)}$$

As a final step to arrive at the formula of Wilson and colleagues, let us rename our free parameter $b_{EV}$ to $\frac{1}{\sigma_{d}}$, parameter $b_{SB}$ to $\frac{B}{\sigma_{d}}$, and $b_{U}$ to $\frac{\alpha}{\sigma_{d}},$. We can thus write

$$P_{right}=\frac{1}{1+exp\left( \frac{R_{\boldsymbol{right}}-R_{\boldsymbol{left}}+\alpha{(I}_{\boldsymbol{right}}-I_{\boldsymbol{left}})+B}{\sigma_{d}} \right)}$$

which is the formula used by Wilson and colleagues in their paper. In other words, by dividing our original formula by the coefficient $b_{EV}$, we factor out this parameter, which represents random exploration. Thus, our model and the model used by Wilson and colleagues are identical (apart from the additional terms we included). Specifically, by dividing by $b_{EV}$ we can obtain parameter estimates that equate the ones obtained by Wilson and colleagues. The terms we included in our formula that were not in the original formula used by Wilson and colleagues allow us to control for an additional repetition bias, and test how parameters change when the choice horizon changes.

Introduction

We found that rhesus monkeys engaged in strategic exploration by decreasing their reliance on expected values (*random* exploration) when it was useful for the future (long horizon) and that active sampling was the only way to obtain information (partial feedback). Neurally, we found prefrontal strategic exploration signals in the ACC/MCC and dlPFC. However, we did not find a significant modulation by the horizon and feedback type of the effect of uncertainty (*directed* exploration) on choices.

Results

In other words, animals strategically modulated the degree to which they used *random* exploration both depending on the horizon length and feedback type (Fig S3A).

We also ran a second model that used the number of available information (which is mathematically equivalent to the model used by Wilson and colleagues (12)) rather than uncertainty, and found identical results, both for the effects on expected value and the absence of an effect on the sensitivity to the number of available information (Fig S3B and see Fig S4 for full model fit). Therefore, uncertainty did not play a key role in strategic exploration in our task. This indicates that our macaques did not use *directed* exploration to strategically guide their choices (Fig S3).

Discussion

However, contrary to human behavior, our monkeys only adapted by reducing their reliance on expected value (i.e., exploitative value) on choices, which corresponds to the degree to which they use *random* exploration. Humans also increase their preference for the most uncertain option when exploration is useful for the future, which has been referred to as *directed* exploration (12).

**Fig. S3.** Equivalency between our linear regression and the framing in terms of *random* and *directed* exploration. (**A**) With the uncertainty regressor. We find that monkeys modulate their sensitivity to the expected value depending on the horizon and the feedback type, which is equivalent to the *random* exploration parameter, the softmax noise, which is the inverse of the expected value regressor. However, we find no modulation of the uncertainty by the horizon nor the feedback type, which is equivalent to the *directed* exploration parameter, the uncertainty bonus, which is the uncertainty regressor divided by the expected value regressor. (**B**) Same as A but with the number of available information rather than the uncertainty. Error bars indicate standard deviation.

In their original model, Wilson et al. (2014) included regressors for expected reward, information bonus and side-bias. In the model we used we replaced information bonus by uncertainty, and additionally also controlled for a repetition-bias. Using information bonus rather than uncertainty in the model also does not reveal effects of information bonus on feedback type or horizon and the effects on expected value are identical. In line with the reviewer’s request, we also now report the results of this model in which we replaced uncertainty (Fig S1) by an information bonus in Fig S4.

We used a Bayesian model to quantify expected value and uncertainty on each trial, rather than using an information bonus like Wilson et al. (2014), to account for the differences between our implementation of the task in macaques. In the original task reward was displayed as points ranging from 1 to 100, which was sampled from Gaussians with a mean of either 40 or 60 and a standard deviation of 8. By contrast, in our task each reward translated to a unique number of drops of juice (ranging from 0 to 10), which were sampled from Gaussians with means between 3 and 7 and standard deviations of 1.5. As a result of this change, the range of possible rewards was much narrower in our task. Additionally, our monkeys were overtrained on the task, so they were very familiar with the reward variations. Because of the narrow reward range in our task, some rewards were more informative than others. This figure shows that after a very small or a very high reward, the uncertainty is much smaller than after a medium reward.

Consider a trial during which 3 samples are displayed on one side and 1 on the other side. If the 3 samples were all indicating a very high reward, then the underlying mean on this trial is likely 7, with a narrow uncertainty. By contrast, if the rewards were sampled around 5, then the mean would likely be 5 but with a broader uncertainty (because of the ceiling effect). If we had simply used an information bonus, then both of these example trials would be quantified the same way, but our Bayesian model accounted for potential differences. Thus, we hoped that this model would allow us to tease out some stronger behavioral effects of uncertainty, which unfortunately was not the case as we found no effects of uncertainty on feedback type or horizon.

Our results are exactly the same if we use information rather than uncertainty. We have added the full model fit with information rather than uncertainty and mention this in the Result section.

We ran a second model that used the number of available information (which is mathematically equivalent to the model used by Wilson and colleagues (12)) rather than the uncertainty and found identical results, both for the effects on expected value and the absence of effect on the sensitivity to the number of available information (Fig S3B and see Fig S4 for full model fit).

**Fig. S4.** The same model as in Supplementary Figure 1 but only using the number of available information on each side rather than the uncertainty. All conventions are the same as in Supplementary Figure 1.

For the third point, we have added the following paragraph in our discussion to elaborate on the lack of modulation of the sensitivity to the uncertainty by the horizon and the feedback:

Our results can also be compared to recent work in monkeys showing that monkeys choose novel options until they have an estimate of its value compare to other options (7–9), which can be interpreted as exploration for uncertainty reduction. Here, we found that monkeys did not seek to reduce overall uncertainty. This perhaps is because the number of reward samples was negatively correlated with uncertainty, and thus no option was novel in the sense that there was no information about it. Uncertainty-relate behavior was not modulated by the feedback type nor the horizon, hinting that uncertainty (rather than novelty) was not a driver of strategic exploration. This suggests species-specificity in exploratory strategy. In the future, a variation of our task could be used to test the effect of novelty on strategic exploration by offering zero information about one option.

2. The partial and complete feedback condition comparisons is problematic in isolating processing of counterfactual feedback. This is because in the complete feedback sessions where the feedback is always provided during the forced and free choice trials for both options, the counterfactual feedback is confounded with complete (i.e. equal) information, whereas in the partial feedback sessions the amount of information provided is either equal or unequal. Instead of a direct comparison between the partial and feedback sessions, the authors should be contrasting a randomly sampled subset of trials from the complete feedback sessions with trials in which equal amounts of partial feedback were provided about the two cues in the observation phase. Ideally, the same exact feedback would be provided but I recognize this might be impossible. This contrast and the identified neural activity could then be compared to the currently missing contrast of equal and unequal feedback in the

partial feedback sessions, to better isolate the effects due to counterfactual feedback versus uncertainty.

We believe this comment, at least as it relates to the behavioral analysis, is based on a misunderstanding. For Fig 2 we only use first trials, which means everything about the animals’ experiences at that point in the sequence is identical between conditions. Indeed, the complete feedback is not provided in the observation phase (forced choice) trials. In the complete feedback conditions, we also have 50% of equal information (2/2) and 50% of unequal information (1/3 or 3/1) trials. The amount of information is therefore equivalent in the partial and complete feedback conditions. In fact, partial and complete feedback first trials are undisguisable from each other on the first trial. The type of feedback was fixed during a session and only the type of feedback a monkey got on the previous trial could tell him the session type. This design allowed us to contrast these two conditions on the first choice only. This also means no differences in behavior can be due to differences in experience based on feedback type. Instead, our interaction effect reflects a genuine difference in behavioral approach in the animals.

We have added the following sentence to clarify this for the reader.

Introduction

Second, we manipulated the contingency between choice and information by varying the type of feedback monkeys received after their active choices (the observation phase was identical for partial and complete feedback conditions).

Fig 1 caption

(D) We also modulated the contingency between choice and information by including different feedback conditions. In the partial feedback condition monkeys only receive feedback for the chosen option. In contrast, in the complete feedback condition they receive feedback about both options after active choices (not in the observation phase).

Results

Crucially, there was no difference in the visual inputs between the partial and the complete feedback condition, as the nature of the feedback was not cued and fixed for blocks of sessions and monkeys only received the counterfactual feedback after an active choice (not in the observation phase).

The reviewer points out that we cannot contrast the partial and complete feedback conditions in the subsequent choices, meaning all other choices than the first one. Indeed, after the first choice, the amount of information provided is greater in the complete than the partial feedback condition - and indeed we can only study the effect of the counterfactual feedback when it is provided, in the complete feedback condition. We are fully aware of this and after the first choice, we do not contrast the partial and complete feedback conditions anymore but study them separately, with an emphasis on the more interesting session type for us when it comes to counterfactual learning: the complete feedback condition. Thus, Fig 5 is only related to the complete feedback condition and it is not a contrast with the partial feedback condition. We have expanded our description as follow:

Introduction

Complementing this activity at the time of decision, in the complete feedback condition, we found overlapping chosen and unchosen outcome prediction error signals in the OFC, at the time of receiving the outcome.

Results

After the first choice, the sequences of events played out differently in the partial and complete feedback conditions, therefore we analyzed each dataset separately in regard to feedback.

We then turned to the complete feedback condition, in which we simultaneously presented the outcome of the chosen and the unchosen after the first active choice, in order to examine the neural substrates involved in learning about counterfactual feedback and the extent to which they overlap with learning about chosen feedback.

We have also made this clear in our new description of the activation related to the uncertainty about the chosen option.

We also found a clear representation of the uncertainty about the chosen on the first choice (when the magnitude of the uncertainty about the chosen option is equivalent in the partial and complete feedback conditions as no counterfactual feedback has yet been provided) in the right medial prefrontal cortex (24c and 9m) that extended bilaterally in the frontal pole (10mr) (Fig S4B).

3. The use of one-tailed statistical tests is not defensible and many of the key effects, particularly relating to the horizon manipulations would not survive appropriate corrections for multiple comparisons.

As the reviewers states earlier in their review, our task is closely related to Wilson et al, (2014) except with an additional test for strategic exploration. Therefore, the direction of our expected effect was predetermined. For example, we *could not* have interpreted an increase in use of exploitative value as evidence *for* exploration. As the sidedness of a statistical effect should reflect the type of hypothesis, we ran a one-sided test as doing otherwise would have biased us against significance based on our prior hypothesis. Equally, doing a 2-tailed T-test in the case of the horizon manipulations would imply that the other direction than the one we expected would have been a positive result. Using up 1-tail-t-test guarantees that only the direction that we expected can lead to a positive result.

Additionally, to Wilson et al. other works speaks towards the difference between the complete and partial feedback, it has been shown that providing the complete feedback decreases decision noise (Findling et al. 2020) and improves learning (Palminteri et al., 2015, 2017; Bavard et al., 2018; Pischedda et al. 2020) both of which are consistent with an increased sensitivity to the expected value. We did not make this last part clear in the manuscript, so we have added these references in the introduction. Furthermore, had we found more exploration in the complete feedback condition, it would have been disingenuous to call this ‘strategic exploration’, which again warrant the use of a one-sided test.

The last effect is the interaction between the feedback type and the horizon. **The direction of the interaction is at the heart of our design (Fig 1A).** Indeed, we set up this study specifically to test whether the prospective effect the horizon has on exploration would be annihilated or at least diminished if there was no contingency between the choice and the feedback. This would show that it is obtaining this feedback that drives the exploration on the first choice in the long horizon. If we had observed no effect of feedback, this would have meant that monkeys use a heuristic that is to increase exploration in the long horizon but independently of actually getting the information. If we had found the opposite pattern: that monkeys explore more in the long horizon than the short horizon when both feedbacks are given, this would have meant that monkeys somehow self-sabotage in the complete feedback condition, and certainly this would not have been a strategic behavior in the sense of an optimal policy. Therefore, there was only one direction of the effect that makes sense here and only tested for it.

As in Wilson and colleagues’ original study, we manipulated whether the information could be used for future choices by changing the choice horizon (12). By comparing exploration in both conditions, we could test whether the animals reduced their reliance on value estimates (*random* exploration) and increased their preference for more uncertain options (*directed* exploration) when gathering information was useful for future choices in the long horizon (12). In addition, we manipulated the contingency between the choice and the information by varying the type of feedback that monkeys received. In the complete feedback condition, information was freely available, and we could probe whether monkeys decreased their exploration compared to the classic partial feedback condition. In humans, providing the complete feedback decreases decision noise (19) and improves learning (20–23) both of which are consistent with reduced exploration. A strategic explorer would only actively explore – and forgo immediate rewards – when it is useful for the future (long horizon) and that it is the only way to obtain information (partial feedback). In addition to behavioral data, neural data were collected using fMRI to probe the neural substrates of strategic exploration. Our analysis was focused on regions previously identified in fMRI studies on reward valuation and cognitive control in monkeys (24–29). Finally, we took advantage of the different feedback conditions to explore how monkeys update their expectations based on new information. Specifically, we investigated the behavioral and neural consequences of feedback about the outcome of their choice and – in the complete feedback condition – on counterfactual feedback from the alternative.

Additionally, we could have been clearer in the explanation of our behavioral analysis. Throughout the manuscript we use a single model that contains all the regressors of interests and confounds for the first choice and a single model for the subsequent choices. We therefore do not need to correct for multiple comparisons, and we have highlighted this further (as each regressor is not an equivalent test for or against the hypothesis which would merit a multiple comparison correction such as Bonferroni). We have added the following sentence to clarify this point.

To test this hypothesis, we ran a single logistic regression predicting responses during first choices in the partial and complete conditions with the following regressors: the expected value according to our Bayesian model, the uncertainty according to our Bayesian model, the horizon (short/long), and the interactions of expected value and uncertainty with horizon. In the same model, we added two potential biases, a side bias and tendency to repeat the same action.

We investigated the determinants of these effects by performing a single logistic regression for all non-first choices with the following regressors: the expected value and uncertainty during the observation phase (which served as a baseline for subsequent choices), the change in these baselines as new information was revealed as they progressed through the horizon. We also added in the same model three potential biases in choices: a side bias, the tendency to repeat the same action, and a bias for choosing the option most often chosen (see Fig S5 for full model fit).
