# Supplementary material for: Neural responses in macaque prefrontal cortex are linked to strategic exploration
Source: PLoS Biol. 2023 Jan 30;21(1):e3001985. doi: 10.1371/journal.pbio.3001985 (PMC9910800; doi:10.1371/journal.pbio.3001985)
Supplement: Attachment — Submitted filename: Response_to_editor_final.docx [file pbio.3001985.s011.docx]

Dear Dr. Dickson,

Firstly, we would like to thank you and the reviewers for their positive assessment of our work, and we are delighted that you found our responses adequate. Please find below the changes we did to the manuscript. We have highlighted textual changes in the manuscript in red, reviewer’s questions are in grey and our replies here in blue.

Based on the reviews and on our Academic Editor's assessment of your revision, we are likely to accept this manuscript for publication. Given that our readership might have similar questions to those raised by the reviewers, we would like you to do some rewriting of your manuscript to incorporate the point-by-point responses you'd provided to Reviewer 1 into the body of your manuscript.

We agree with Dr. Dickson that readers might have similar questions as Dr. Chang. We will therefore publish the response to the reviewers alongside our manuscript. We had taken the position to include significant results that were found following Dr. Chang’s questions and that strengthen the manuscript, but not the non-significant results. We propose to leave non-significant results to the Response to reviewers. In particular, some of the analyses that Dr. Chang suggested were exploratory in nature, in the sense that we didn’t have any hypotheses to support them and/or our data was not well suited to answer them. We detail below which analyses we propose to include or not and why.

*Questions 1, 4, 6 and 7* were addressed in the main text.

*Question 2: Strategic decisions frequently have idiosyncratic components as different monkeys may employ slightly different strategies for solving the same task even when their overall strategies might converge on average. It might be thus worthwhile to examine if there were individual differences in strategies over the course of a session or the course of the entire data collection (e.g., were these decision variables stationary or non-stationary?). Given that there is evidence of individual differences in Supp Figs 1-3, it would be informative to go deeper into how such individual differences might be reflected potentially differently across ACC/MCC, dlPFC, and OFC BOLD activations with respect to their functional roles suggested in this manuscript.*

We have added the following description of the effect of trial number and session number in the Result section.

Finally, we checked whether the decision variables were stable over trials and across sessions. First, in the above regression model, we added interaction terms with the trial number. We found no significant interaction with our regressors of interest (expected value, expected value interaction with horizon, uncertainty and uncertainty interaction with horizon). Second, we fitted each session separately and looked for a linear trend in the session number. We found no linear nor clear trend for the regressors over sessions. Overall, we found no evidence that monkeys’ decision variables changed throughout the recording.

We have additionally highlighted in the manuscript that the posteriors for the model fits are accessible in our data repository. The last part of question 2 is an exploratory analysis of brain-behavior correlations. We do not want to give the readers the impression that we tried our every possible brain-behavior correlation to find significant results. We did perform these exploratory analyses based on the reviewer’s question but none of them were significant. Given that the results were not significant, they are extremely hard to interpret. We would prefer not to present them in the main text. However, if Dr. Dickson feels that this is critical, we could add the following paragraph:

“Based on a reviewer’s request, we conducted additional exploratory brain behavior correlations between our regressors and the activation in our ROIs. This was aimed to find any individual differences in the activations that we describe. We however found no significant relationship between the behavioral sensitivity to the expected value across conditions and the neural activations in our ROIs.”

*Question 3: In P. 9, "However, we found no statistically reliable difference in the sensitivity to the uncertainty across the experimental conditions. Therefore, uncertainty did not play a key role in strategic exploration in our task." I am a bit surprised that there were no clear effects of uncertainty in monkeys' behaviors, as reducing uncertainty should be a major functional driver of decisions that are central to exploration. Unless I missed it, I don't see a place where the authors examined the effects of uncertainty at the neural activation level and how it might have influenced the neural representation of strategic decision variables in the brain regions examined. It would be surprising if uncertainty (variance) played no role in ACC/MCC, dlPFC, or OFC activations on signaling expected value or chosen/unchosen outcomes. If there are any effects, these modulations might further provide neural mechanistic insights into the computations performed by these brain regions for explore vs. exploit decisions. Either way, I suggest the authors discuss in some detail the effects of uncertainty (behavioral and neural) in the Discussion in relation to the main findings.*

We have added a description of the neural correlate of uncertainty that corresponded to a behavioral effect. We left out from the main manuscript the description of the neural correlates of uncertainty that did not correspond to a behavioral effect. When looking at uncertainty in brain, we only found a significant cluster was in the chosen uncertainty in partial vs. complete feedback, which showed more activity related to the uncertainty in the partial feedback in a cluster in the right somatosensory cortex 1-2 and extending in the claustrum. We find it very hard to interpret this result as this difference does not support any behavioral change, nor does it appear in a network known to influence decision-making. Therefore, we do not think it should be included in the main text. As for question 2, we performed exploratory analyses looking at the relationship between uncertainty sensitivity and the activations that we found but none of them were significant. We find this hard to interpret given that they are not significant, so we would prefer not to include it in the main text. However, if Dr. Dickson feels that this is critical, we could add the following paragraph and display the fMRI results in a supplementary figure:

“Based on a reviewer’s request, we looked for any uncertainty related activity that differed between our experimental conditions, even if they did not support a behavioral change. We found a significant cluster of activity for the chosen uncertainty in partial vs. complete feedback (right somatosensory cortex 1-2 and extending in the claustrum). We also conducted additional exploratory brain-behavior correlations between our regressors and the activation in our ROIs. This was aimed at looking if the uncertainty plays a role in the activations we described. We found however no significant relationship between the behavioral sensitivity to uncertainty and the neural activations in our ROIs.”

*Question 5: Differentiated or common roles of different brain regions tracking decision variables were interesting. Were there any different functional connectivity patterns among different brain regions for exploration vs. exploitation and in long vs. short horizons? Such network-level analyses may provide additional support on how different brain regions work together to compute strategic choices.*

As stated in the response to reviewers, we examined connectivity patterns between our ROIs using PPIs. Unfortunately, our results remained inconclusive and were hard to interpret so we did not include any of them in the main manuscript. The pipeline that we used is not well suited for the type of analysis that the reviewer requested so including a long description of a non-results would dilute the effects of interest that we report in the paper.

We also ask that you consider a title change to something like:

The macaque prefrontal cortex supports strategic exploration (If you don't feel this is too strongly worded)
OR
Neural responses in macaque prefrontal cortex are linked to strategic exploration

We thank the editor for this suggestion. We have changed the title to: “Neural responses in macaque prefrontal cortex are linked to strategic exploration”

Please also make sure to fully address the data and other policy-related requests at the bottom of this email. Failure to do so will result in delays in moving your submission forward.

As you address these items, please take this last chance to review your reference list to ensure that it is complete and correct. If you have cited papers that have been retracted, please include the rationale for doing so in the manuscript text, or remove these references and replace them with relevant current references. Any changes to the reference list should be mentioned in the cover letter that accompanies your revised manuscript.

We expect to receive your revised manuscript within two weeks. 

To submit your revision, please go to <https://www.editorialmanager.com/pbiology/> and log in as an Author. Click the link labelled 'Submissions Needing Revision' to find your submission record. Your revised submission must include the following:
-  a cover letter that should detail your responses to any editorial requests, if applicable, and whether changes have been made to the reference list
-  a Response to Reviewers file that provides a detailed response to the reviewers' comments (if applicable)
-  a track-changes file indicating any changes that you have made to the manuscript. 

NOTE: If Supporting Information files are included with your article, note that these are not copyedited and will be published as they are submitted. Please ensure that these files are legible and of high quality (at least 300 dpi) in an easily accessible file format. For this reason, please be aware that any references listed in an SI file will not be indexed. For more information, see our Supporting Information guidelines:
<https://journals.plos.org/plosbiology/s/supporting-information>  

*Published Peer Review History*
Please note that you may have the opportunity to make the peer review history publicly available. The record will include editor decision letters (with reviews) and your responses to reviewer comments. If eligible, we will contact you to opt in or out. Please see here for more details:
<https://blogs.plos.org/plos/2019/05/plos-journals-now-open-for-published-peer-review/>

*Press*
Should you, your institution's press office or the journal office choose to press release your paper, please ensure you have opted out of Early Article Posting on the submission form. We ask that you notify us as soon as possible if you or your institution is planning to press release the article.

*Protocols deposition*
To enhance the reproducibility of your results, we recommend that if applicable you deposit your laboratory protocols in [protocols.io](http://protocols.io), where a protocol can be assigned its own identifier (DOI) such that it can be cited independently in the future. Additionally, PLOS ONE offers an option for publishing peer-reviewed Lab Protocol articles, which describe protocols hosted on [protocols.io](http://protocols.io). Read more information on sharing protocols at <https://plos.org/protocols?utm_medium=editorial-email&utm_source=authorletters&utm_campaign=protocols>


Please do not hesitate to contact me should you have any questions.

Sincerely,

Kris

Kris Dickson, Ph.D., (she/her)
Neurosciences Senior Editor/Section Manager,
[kdickson@plos.org](mailto:kdickson@plos.org),
PLOS Biology

------------------------------------------------------------------------
ETHICS STATEMENT:

Please update your ethics statement with the required additional details as outlined in
<https://journals.plos.org/plosbiology/s/animal-research#loc-non-human-primates>

Specifically, non-human primate studies must be performed in accordance with the recommendations of the Weatherall report “The use of non-human primates in research”. Manuscripts describing research involving non-human primates must include details of animal welfare, including information about housing, feeding, and environmental enrichment, and steps taken to minimize suffering, including use of anesthesia and method of sacrifice if appropriate.

We have added the following description:

Animals were kept on a 12-hour light dark cycle, with access to water for 12-16 hours on testing days and with ad libitum water otherwise. Feeding, social housing and environmental enrichment followed guidelines of the Biomedical Sciences Services of the University of Oxford. All procedures were conducted under licenses from the United Kingdom (UK) Home Office in accordance with The Animals (Scientific Procedures) Act 1986 and the European Union guidelines (EU Directive 2010/63/EU).

------------------------------------------------------------------------
DATA POLICY:

You may be aware of the PLOS Data Policy, which requires that all data be made available without restriction: <http://journals.plos.org/plosbiology/s/data-availability>. For more information, please also see this editorial: <http://dx.doi.org/10.1371/journal.pbio.1001797> 

We appreciate the deposition of your data on Gitfront but note that we cannot accept sole deposition of data to non-static site (e.g. no personal sites and generally no institutional sites). (<https://journals.plos.org/plosbiology/s/data-availability>). We require deposition of all summary data to a static site, like Zenodo, FigShare or OSF. GitHub and similar sites can be used for depositing code however.

Note that we do not require all raw data to be deposited on such a static site. Rather, we ask that all individual quantitative observations that underlie the data summarized in the figures and results of your paper be made available in order to allow our readership to reproduce the figures in your paper:

1) Please ensure that these data files are invariably referred to (in the manuscript, figure legends, and the Description field when uploading your files) using the following format verbatim: S1 Data, S2 Data, etc. If using excel, multiple panels of a single or even several figures can be included as multiple sheets in one excel file that is saved using exactly the following convention: S1_Data.xlsx (using an underscore).

2) Please also provide the updated accession code so that we may view your data before publication.

3) Please ensure that you provide the individual numerical values that underlie the summary data displayed in the following figure panels as they are essential for readers to assess your analysis and to reproduce it:
Fig2A-D; Fig3A-E; Fig4B,D,E; Fig5B-D
Supplemental figures: S1A,B; S2A-C; S3A,B; S4A-C; S5A-C;

NOTE: the numerical data provided should include all replicates AND the way in which the plotted mean and errors were derived (it should not present only the mean/average values).

4) Please also ensure that figure legends in your manuscript include information on where this underlying data can be found (e.g. “The underlying data supporting Fig X, panel Y can be found in file Z.”), and please also ensure that your supplemental data file/s has a legend.

5) Please ensure that your Data Statement in the submission system accurately describes where your data can be found.

Data and code to reproduce the figures can be found at <https://doi.org/10.5281/zenodo.7464572>. This statement was added below each figure.

------------------------------------------------------------------------
DATA NOT SHOWN?

- Please note that per journal policy, we do not allow the mention of "data not shown", "personal communication", "manuscript in preparation" or other references to data that is not publicly available or contained within this manuscript. Please check your submission to ensure no such statements are included. If there are such statements, either remove mention of these data or provide figures presenting the results and the data underlying the figure(s).

No such statements were included.

------------------------------------------------------------------------

Reviewer remarks:

Do you want your identity to be public for this peer review?

Reviewer #1: Yes: Steve W. C. Chang

Reviewer #2: No

Reviewer #3: No

Reviewer #1: The authors did a very thorough job at addressing all of my points by 1) performing a number of new analyses to support their responses, 2) adding new information on the manuscript, and 3) modifying their interpretations more broadly in the context of explore vs. exploit decision-making. Their responses were detailed and constructed based on evidence from their data (even though not included in the manuscript). I have no more comments.


Reviewer #2: The authors have addressed my concerns.  I have no further comments.


Reviewer #3: The authors have have satisfactorily addressed my previous concerns.
